# Thermodynamic equilibrium between locally excited and charge-transfer states through thermally activated charge transfer in 1-(pyren-2′-yl)-*o*-carborane†

**DOI:** 10.1039/d1sc06867a

**Published:** 2022-04-19

**Authors:** Lei Ji, Stefan Riese, Alexander Schmiedel, Marco Holzapfel, Maximillian Fest, Jörn Nitsch, Basile F. E. Curchod, Alexandra Friedrich, Lin Wu, Hamad H. Al Mamari, Sebastian Hammer, Jens Pflaum, Mark A. Fox, David J. Tozer, Maik Finze, Christoph Lambert, Todd B. Marder

**Affiliations:** Frontiers Science Center for Flexible Electronics, Xi'an Institute of Flexible Electronics (IFE), Northwestern Polytechnical University 127 West Youyi Road Xi'an Shaanxi China iamlji@nwpu.edu.cn; Institut für Anorganische Chemie and Institute for Sustainable Chemistry & Catalysis with Boron, Julius-Maximilians-Universität Würzburg Am Hubland 97074 Würzburg Germany todd.marder@uni-wuerzburg.de; Institut für Organische Chemie, Julius-Maximilians-Universität Würzburg Am Hubland 97074 Würzburg Germany christoph.lambert@uni-wuerzburg.de; Department of Chemistry, University of Durham South Road Durham DH1 3LE UK basile.curchod@bristol.ac.uk; Department of Chemistry, College of Science, Sultan Qaboos University PO Box 36, Al Khoudh 123 Muscat Sultanate of Oman; Experimentelle Physik VI, Julius-Maximilians-Universität Würzburg Am Hubland 97074 Würzburg Germany

## Abstract

Reversible conversion between excited-states plays an important role in many photophysical phenomena. Using 1-(pyren-2′-yl)-*o*-carborane as a model, we studied the photoinduced reversible charge-transfer (CT) process and the thermodynamic equilibrium between the locally-excited (LE) state and CT state, by combining steady state, time-resolved, and temperature-dependent fluorescence spectroscopy, fs- and ns-transient absorption, and DFT and LR-TDDFT calculations. Our results show that the energy gaps and energy barriers between the LE, CT, and a non-emissive ‘mixed’ state of 1-(pyren-2′-yl)-*o*-carborane are very small, and all three excited states are accessible at room temperature. The internal-conversion and reverse internal-conversion between LE and CT states are significantly faster than the radiative decay, and the two states have the same lifetimes and are in thermodynamic equilibrium.

## Abstract 2

Reversible conversion between excited-states is key to many photophysical phenomena. We studied the equilibrium between LE and CT states by time-resolved and temperature-dependent fluorescence, fs- and ns-transient absorption, and LR-TDDFT calculations.

## Graphical abstract

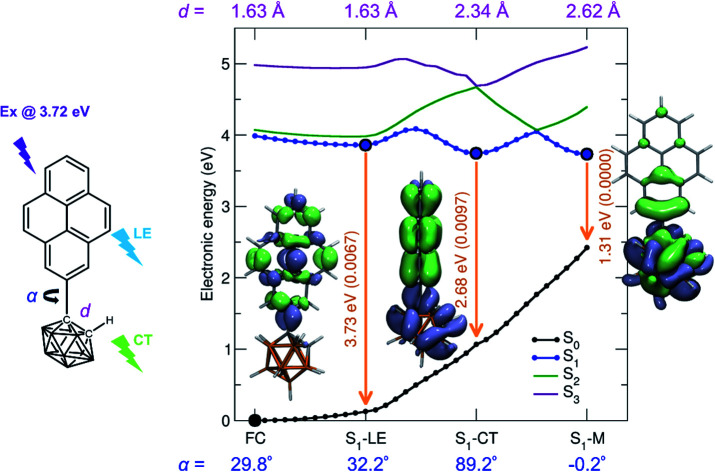

## Introduction

Interconversion between excited states is crucial in many photophysical processes. In most cases, these interconversions are not reversible, as the energy differences between the initial and final states are usually so large that the reverse process is thermodynamically forbidden, and lifetimes of the lower excited states are too short to allow for an efficient back reaction. Thermally activated delayed fluorescence (TADF)^1,2^ is one important example of reversible excited state interconversion, in which the reverse intersystem crossing (RISC) from a dark T_1_ state to a slightly higher lying bright S_1_ state is thermodynamically allowed at room temperature. In this case, the RISC is usually much slower than radiative decay from the S_1_ state, so a short lifetime (∼ns) and a long lifetime (∼μs) emission component are detected at the fluorescence band (E-type fluorescence).^1^ As the S_1_ state decays very quickly, but drains the T_1_ state slowly, the long lifetime is strongly affected by the RISC rate (*k*_−isc_).

However, the ISC rate (*k*_isc_) from S_1_ to a triplet state should be of the same order of magnitude as the fluorescence decay rate, otherwise the triplet state will not be populated after photo excitation. As *k*_isc_/*k*_−isc_ depends on the S–T energy gap (Δ*G*) between S_1_ and T_1_, *k*_−isc_ can be comparable with the fluorescence decay rate when the S–T gap is small enough, leading to the possibility of a thermodynamic equilibrium between the S_1_ and T_1_ states. However, this is hard to observe as the triplet is a dark state and the fluorescent S_1_ state can have only one lifetime if *k*_−isc_ is very fast. In this case, the system is very close to that in which a molecule has a fluorescent singlet excited state with one or more dark singlet excited states that are close in energy, as internal conversion between the excited states is very fast. An example which we could use to study the excited state equilibrium process is a dual-fluorescent dye, for which two emissive singlet states can be in equilibrium.

Based on their dual fluorescence properties, donor–π–acceptor compounds with bright locally excited (LE) and intramolecular charge transfer (CT) states have been examined as models for studying CT processes.^3–6^ For example, dimethylamino benzonitrile (DMABN) has a highly emissive CT state, which is well known as a twisted intramolecular charge transfer (TICT) state,^6,7^ because a significant amount of CT takes place during the twisting of the dimethylamino group in the excited state.^6–9^ A fast CT process may cause quenching of the LE emission. To achieve dual emission from both LE and CT (TICT) states, much effort has been devoted to reducing the CT rate (*k*_CT_, ∼10^11^ s^−1^) to make the LE and CT emissions co-exist kinetically, such as suppression of Kasha's rule by applying viscous surroundings to a TICT luminophore, which has been employed to enhance the fluorescence of some molecular rotors.^10,11^ In this case, the CT rate (*k*_CT_, ∼10^11^ s^−1^), which is similar to the internal conversion rate, decreases and is comparable to that of the radiative decay rate of the LE states (*k*_r,LE_, ∼10^9^ s^−1^) because viscous media inhibit the formation of a twisted structure and thus slow down the CT process.^12–14^

LE and CT states can also be in thermodynamic equilibrium,^15–18^ but both *k*_CT_ and *k*_−CT_ (the rate of LE ← CT) have to be much faster than the radiative decay rates of both the LE and CT states. When *k*_CT_ and *k*_−CT_ are significantly faster than the radiative decay rates, thermodynamic equilibrium can be assumed to be adiabatic at any time during the decay, so the experimental fluorescence lifetimes of the LE and CT emissions must be identical (*vide infra*). This indicates that an apparent one-band emission with a monoexponential lifetime could also be an overlay of several emissions. To achieve fast *k*_CT_ and *k*_−CT_ and thermodynamic equilibrium, the barriers for the molecular reorganization during the internal conversion as well as the energy gap between LE and CT states must be small. Dyes with well-separated fluorescence bands from LE and CT states, respectively, which are in thermodynamic equilibrium, are rare.^15^ This is because the LE and CT states have to lie at similar energy levels to achieve thermodynamic equilibrium, which usually leads to the overlap of the LE and CT fluorescence emissions.

**Fig. 1:**
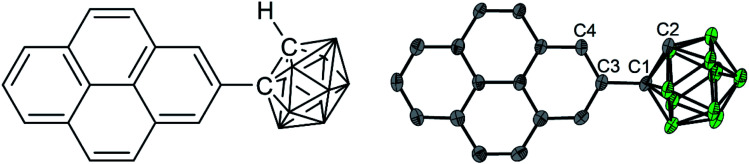
Chemical structure (left) and solid-state geometry determined by single crystal X-ray diffraction of 1 (right, one of four symmetry-independent molecules is shown). Selected distances (Å) and dihedral angles (°) of the four molecules: C1–C2 1.647(6)/1.638(6)/1.640(6)/1.647(6); C2–C1–C3–C4/C16 39.9(6)/−24.2(6)/30.6(6)/−33.5(6). Atom colours: gray (carbon), green (boron). Hydrogen atoms are omitted for clarity.

As a target molecule for our investigations, we employed a simple dyad, namely 1-(pyren-2′-yl)-*o*-carborane 1 (Fig. 1), which displays an extremely low energy barrier for “twisting” (rotation) the pyrene donor with respect to the acceptor moiety, as a model. Monoaryl-*o*-carboranes are high-frequency molecular rotors in which the *o*-carborane rotates rapidly with respect to the C_aryl_–C_carb_ bond (C1–C3 in Fig. 1) because of negligible steric hindrance.^19^ In the excited states, the electron accepting property of *o*-carborane depends on the dihedral angle between the C1–C2 bond and the aryl plane of pyrene; it is weak when the C1–C2 bond is parallel to the aryl group but very strong when the aryl/C1–C2 dihedral angle is large, which allows CT from the aryl to the carborane unit.^19^ This is due to the fact that the main contribution to the LUMO of *o*-carborane arises from the C–C antibonding (σ*) orbital,^19^ which spatially overlaps the π-system of the C-attached aryl moiety when the C1–C2 bond is perpendicular to the aryl plane. These rotamers can result in dual-fluorescence from monoaryl-*o*-carboranes as both the LE and CT states can coexist.^19–29^ Studies of aryl-*o*-carboranes thus far have focused largely on their aggregation-induced emission properties.^19,30–44^ Emissive aryl-*o*-carboranes have been used in organic light-emitting diodes^27,45^ and bioimaging.^46^ Chujo and co-workers reported temperature-dependent dual emissive properties of several Ar-*o*-carboranes (Ar = anthracen-9-yl, 4-diphenylaminophenylene) in solution,^22,47–49^ based on which they estimated the LE–CT energy gaps of these monoaryl-*o*-carboranes to be 2.9–4.6 kJ mol^−1^.^21,48,49^ This allows LE ← CT internal conversion and provides the possibility for thermodynamic equilibrium between the LE and CT states even at room temperature. However, the low fluorescence quantum yields (*Φ* < 0.02) as well as subtle LE : CT band ratio changes as a function of temperature complicated attempts to obtain a full understanding of these systems. In addition, the reported fluorescence lifetime of the LE band is very different from that of the CT band in all of their examples, indicating that adiabatic thermodynamic equilibrium may not have been established.^22,47^ Kang and co-workers have estimated that the ‘photo-induced electron transfer rates’ in *o*-carboranes with carbazole as the donor are *ca.* 8 × 10^9^ s^−1^, based on the fluorescence lifetimes of its LE band.^3,4^ While our study was in progress, Kang's group examined excited-state processes in dual-emissive diaryl-*o*-carboranes in dichloromethane solution by fs-transient absorption spectroscopy,^50^ but their fluorescence quantum yields were very low (≤0.0001 in dichloromethane) and their lifetimes are too short (≤180 ps in dichloromethane) to allow the two excited states to equilibrate.

In this paper, we employed a pyren-2-yl moiety as the aryl group because the fluorescence emissions from 2-substituted pyrenes show intrinsic vibrational structures and long lifetimes,^51–61^ which could provide adequate time to allow the LE and CT states to equilibrate. We studied the photoinduced CT process and the thermodynamic equilibrium between the CT state and the thermally activated LE states by combining temperature-dependent luminescence spectroscopy, fs- and ns-transient absorption spectroscopy, and DFT and LR-TDDFT calculations.

## Results and discussion

### Absorption and emission spectra in hexane

The synthesis of compound 1 is based on our reported synthesis of 2-ethynylpyrene *via* a regioselective CH borylation strategy.^62,63^ Refluxing 2-ethynylpyrene and decaborane (B_10_H_14_) in toluene with acetonitrile as the catalyst gave pure 1 in moderate isolated yield (28%).

**Fig. 2:**
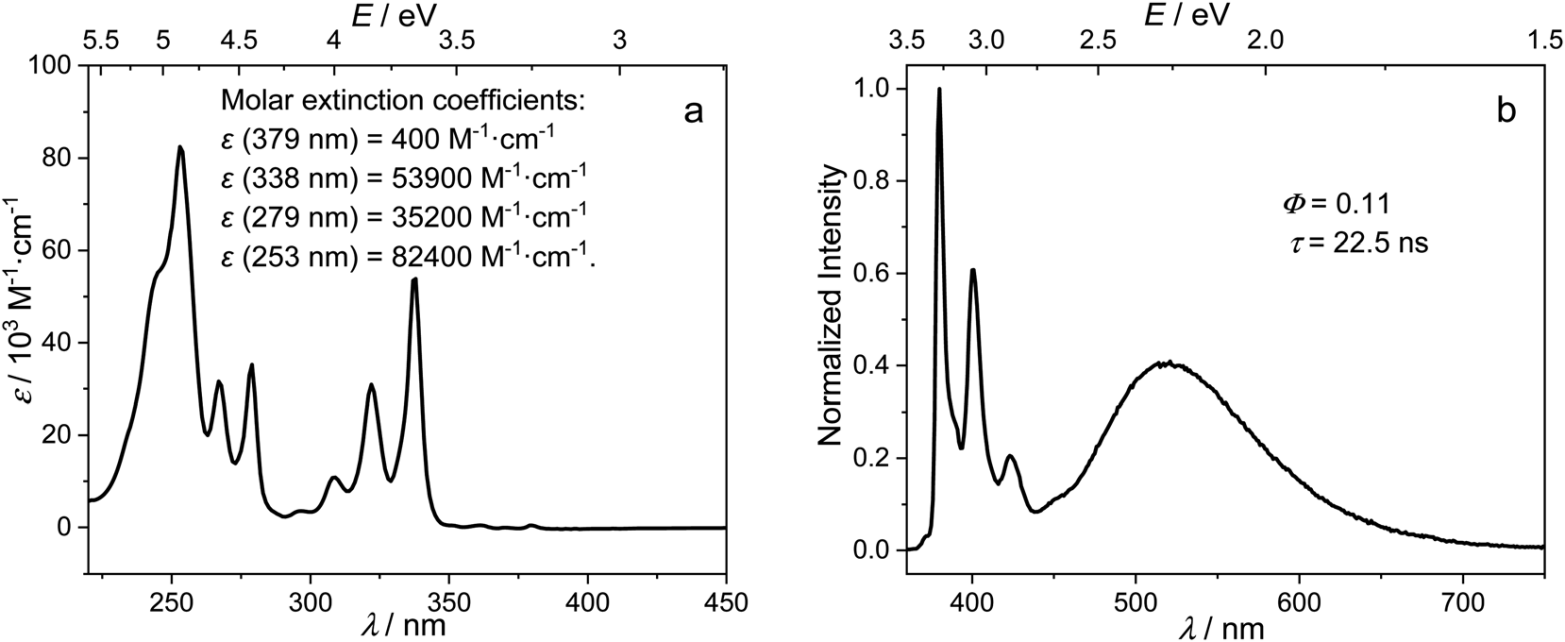
Absorption (a) and normalized emission (b) excitation at 338 nm (3.67 eV) spectra of 1 in hexane at room temperature.

In comparison with pyrene, compound 1 shows a very weak, slightly red-shifted S_1_ ← S_0_ transition (L_b_, 380 nm (3.26 eV)) in the absorption spectrum of its hexane solution (Fig. 2a). Both the fine structure and energy of the S_2_ ← S_0_ transition (L_a_, 338 nm (3.67 eV)) are similar to those of pyrene, because this transition is dominated by the LUMO ← HOMO contribution, which is not affected by the substituents at the 2-position, as the 2-position lies in a nodal plane of both the pyrene HOMO and LUMO.^51–53^

Upon excitation in hexane at 338 nm (3.67 eV), the emission spectrum of 1 (Fig. 2b) shows two intense bands (*Φ* = 0.11 under argon) at room temperature, namely a higher energy (380 nm, 3.26 eV), pyrene-like band with visible vibrational fine structure, which corresponds to the pyrene L_b_ state (hereafter called the LE state because it is localized at the pyrene unit), and a lower energy broad band, which corresponds to the CT state (521 nm, 2.34 eV), according to the photophysics of known aryl-*o*-carboranes and calculations (*vide infra*).^19^ The Stokes shift of the LE band is almost zero, indicating the absence of significant structural changes in the LE state, as confirmed by theoretical calculations presented below. Excitation at 338 nm (3.67 eV) populates the pyrene S_2_ state which relaxes to the pyrene S_1_ state (= LE) *via* very rapid internal conversion. Subsequent charge transfer from the pyrene to the carborane to yield the CT state is accompanied by a significant geometry change. This was confirmed by the emission spectrum of 1 in methylcyclohexane at 77 K, in which the geometry changes are impeded by the frozen matrix, and thus only the LE band is observed (Fig. S1 in the ESI†).

The ratio of the LE and CT bands is not concentration-dependent at concentrations between 10^−6^ to 10^−5^ mol L^−1^ (Fig. S2 in the ESI†), and formation of excimers in hexane at the experimental concentration can thus be excluded. At room temperature under argon, the fluorescence lifetimes (*τ*) of the LE and CT bands, as measured by time-correlated single-photon counting (TCSPC) with an instrument response function of 1.34 ns full-width-at-half-maximum (for decay profiles see Fig. S3 in the ESI†), are the same (*τ*_CT_ = *τ*_LE_ = 22.5 ns) and are typical of the long-lived excited states of 2-substituted pyrenes.^51–53^ The equivalent, single-component lifetimes of the LE and CT states suggest that the bright LE and CT states are in a fast thermodynamic equilibrium, and the reversible internal conversion rates between the LE and CT states, *k*_CT_ and *k*_−CT_, are much faster than all other decay processes and the intersystem crossing rates of the two states; that is:

1 *k*_CT_ ≫ *k*_r,LE_ + *k*_nr,LE_ + *k*_isc,LE_

2 *k*_−CT_ ≫ *k*_r,CT_ + *k*_nr,CT_ + *k*_isc,CT_

The subscripts r, nr, and isc refer to radiative decay, nonradiative decay, and intersystem crossing of the LE and CT state, respectively.

### Temperature-dependent fluorescence spectroscopy

**Fig. 3:**
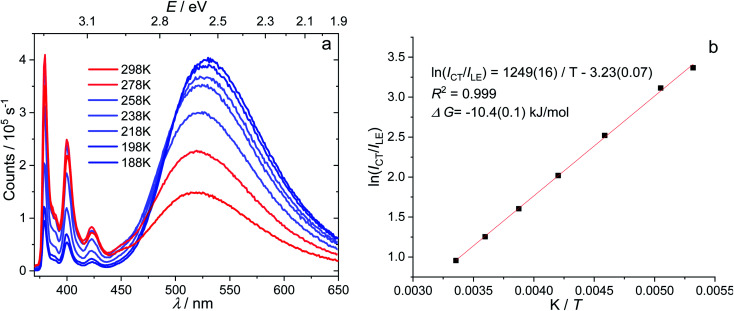
Emission spectra of 1 (*c* = 1 × 10^−5^ mol L^−1^) in hexane at different temperatures (a) and Stevens–Ban plot^64^ of its LE and CT bands (b), where *K* is the equilibrium constant and *T* is the temperature.

Temperature-dependent fluorescence spectroscopy is often applied in studying dual-emissive TICT systems,^65–67^ in most of which the band ratio changes because of the temperature-dependent viscosity of the solvents. In such systems, the viscosity change can slow the twisting and regulate the CT process; thus, the fluorescence band ratio is kinetically controlled. In this work, temperature-dependent fluorescence spectroscopy in hexane and methylcyclohexane was used to study the thermodynamic equilibrium between the LE and CT states of 1. The lifetimes of the LE and CT bands remained identical to each other at all temperatures in the measured range (218–298 K, Fig. S4†), indicating that thermodynamic equilibrium could be reached in a very short time. However, the lifetimes are longer at lower temperatures, probably due to the reduced nonradiative decay rates and increase in the CT state population, the radiative decay rate of which is much slower than that of the LE state (*vide infra*). By cooling a hexane solution of 1 from room temperature to 188 K, the intensity of the LE band decreases while that of the CT band increases, with the LE : CT ratio changing from 1 : 2.6 to 1 : 29 (Fig. 3a). This indicates that the CT state is more populated at lower temperature and has a lower energy than the LE state, according to Maxwell–Boltzmann statistics, but the energy gap between LE and CT states (Δ*G*) must be so small that both states can still be sufficiently populated between 188–298 K. In hexane solution, at any short time Δt after excitation, as *k*_CT_ and *k*_−CT_ are much faster than other processes (eqn (1) and (2)), so 1 can be considered as an adiabatic system in equilibrium. According to Maxwell–Boltzmann statistics, the equilibrium constant (*K*) of molecules in the CT and LE states at temperature *T* is:3
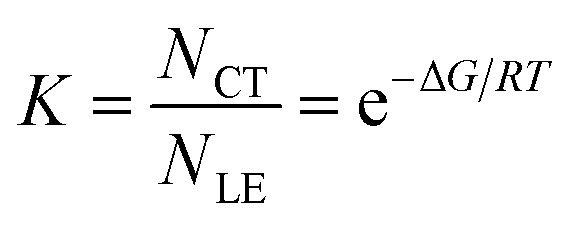
where *N* is the population of each excited state, and *N*_CT_/*N*_LE_ is equal to the concentration ratio [CT]/[LE] in solution. In the steady-state fluorescence measurement, as the concentrations of the excited states are constant, the fluorescence intensity (*I*) is directly proportional to the population of the excited states and the radiative decay rates:

4 *I*_i_ ∝ *N*_i_*k*_i_

The CT/LE band ratio can be written as eqn (5) using eqn (3) for the ratio of *N*_CT_/*N*_LE_:5
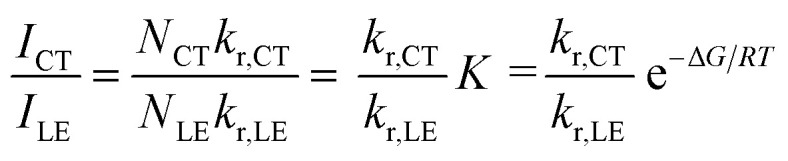
where *k*_r_ is the radiative decay rate and Δ*G* is the energy difference between CT and LE states. Eqn (5) can also be linearized by taking the logarithm on both sides:6
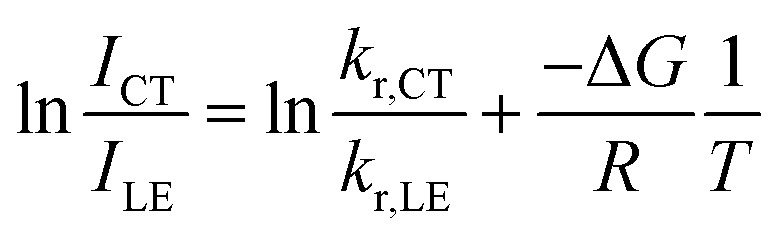


As the entropy difference between the LE and CT states is assumed to be very small (Δ*S* ≈ 0), Δ*G* is equal to Δ*H* and is thus not greatly affected by temperature; *k*_r,CT_ and *k*_r,LE_ are also constants in a certain temperature range. Thus, eqn (6) gives a linear dependence between the logarithm of the band ratio and 1/*T*.

In the temperature-dependent steady-state spectra of 1 in hexane, the plot of ln(*I*_CT_/*I*_LE_) *versus* 1/*T* (Fig. 3b), which is similar to a Stevens–Ban plot,^64^ can be fitted linearly with a regression coefficient of 0.999, in agreement with eqn (6). The slope of the fitted line (−Δ*G*/*R* = 1249 K) reveals that the LE state lies only *ca.* 10.4 kJ mol^−1^ (0.11 eV) above the CT state. This allows the reverse internal conversion from CT to LE states, which is a thermally activated CT process from the carborane back to the pyrene unit. The small energy gap also indicates that the large bathochromic shift of the CT band (0.48 eV from the LE band, calculated from the onset of the CT band (= *E*_0–0_,_CT_) minus the 0–0 transition energy of the LE band, *E*_em,LE,_; see Table 1) is due to the vibrationally excited high energy level of the Frank–Condon ground state 
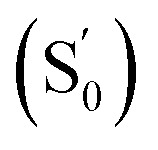
 at the CT geometry, which is 0.81 eV higher than that of S_0_. This makes the thermal population of 
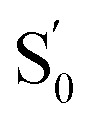
 unachievable at room temperature and is responsible for the absence of a CT band in the absorption and excitation spectra. Temperature-dependent spectroscopy of 1 in methylcyclohexane gives a similar energy gap (Δ*G* = 11.4 kJ mol^−1^ (0.12 eV) between the LE and CT states, see Fig. S5 and S6 in the ESI†).

**Table 1:** Photophysical properties of 1 in various of solvents at room temperature^a^

Solvent	*E*_abs_ (S_1_ ← S_0_)/eV (/nm)	*E*_em,LE_/eV (nm)	*E*_em,CT_/eV (nm)	*E*_0–0,CT_ b/eV	*E*_0–0,St_ c/eV	Δ*E*_LE–CT_/eV
Hexane	3.27 (379)	3.26 (380)	2.34 (521)	2.78	0.48	0.11^d^
Toluene	3.25 (382)	3.24 (383)	1.92 (630)	2.42	0.82	0.45^e^
Chloroform	3.25 (381)	3.25 (382)	1.72 (684)	2.33	0.92	0.55^e^
THF	3.26 (380)	3.25 (381)	1.60 (739)	2.22	1.03	0.66^e^

a Values in parentheses are the directly measured wavelength of absorption/emission maxima. Values not in parenthesis are the absorption/emission maxima with energy as the abscissa axis, which is directly converted using the forumla *E* = *hc*/*λ* for absorption, and with the Jacobian conversion *F*(*E*) = −*F*(*λ*)*hc*/*E*^2^ = −*F*(*λ*)*λ*^2^/*hc* for emission.

b 0–0 transition energy of the CT emission band.

c Stokes shift calculated from the energy difference between the 0–0 transition of the CT emission band and *E*_abs_ (S_1_ ← S_0_).

d Estimated from the Stevens–Ban plot (Fig. 3b) and eqn (3).

e Estimated from Δ*E*_0–0_ (CT) and by assuming that the energy of 
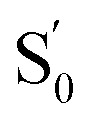
 is the same in all solvents used.

Having assessed the rapid thermodynamic equilibrium in the excited state, we can evaluate the radiative rate constants of the LE and CT states from the common lifetime *τ*_LE_ = *τ*_CT_ = 22.5 ns and the total fluorescence quantum yield (*Φ* = 0.11) to be *k*_r,LE_ = 4.70 (1) × 10^6^ s^−1^ and *k*_r,CT_ = 1.9 (1) × 10^5^ s^−1^.

### Thermodynamic equilibrium in solvents with different polarities

**Fig. 4:**
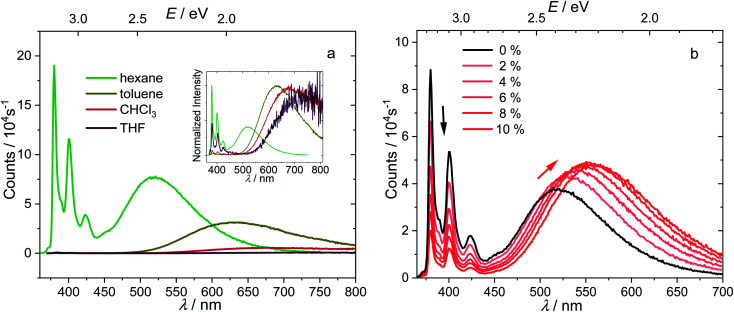
Emission spectra of 1 (*c* = 1.0 × 10^−5^ mol L^−1^, excitation at 338 nm (3.67 eV)) in solvents with different polarities, with the normalized spectra shown in the inset (a), and in hexane with different percentages of added dioxane with fixed concentration (b).

The absorption spectrum of 1 is nearly independent of the solvent, indicating a non-polar ground state. While the energy of the LE band in the emission spectrum does not depend on the polarity of solvent, revealing its pyrene-like local transition character, the CT band is dramatically red shifted (Fig. 4a) in polar solvents, confirming its CT nature. The redshifts of the 0–0 transition are 0.82–1.03 eV (Table 1, the redshifts of CT band maximum in toluene, chloroform, and THF are up to 1.32–1.65 eV), which represent the largest Stokes shifts reported for pyrene-based compounds.^47,51^ This is because stabilization of the CT state by polar solvents enlarges the energy gap between the LE and CT states. In more polar solvents such as toluene, CHCl_3_, and THF, the energy gap between LE and CT states is so large (0.45–0.66 eV, Table 1), that the internal conversion becomes irreversible and the LE state can hardly be thermally repopulated. This significantly quenches the LE band because *k*_CT_ ≫ *k*_r,LE_ and *k*_CT_ ≫ *k*_−CT_, leaving a very weak LE band with a fluorescence quantum efficiency of approximately *k*_r,LE_/*k*_CT_. As the CT emission is also quenched in polar solvents, the LE : CT band ratios appear to be randomly dependent on the solvent, but they are governed more by kinetics (*k*_r,LE_/(*k*_CT_·*k*_r,CT_)) rather than thermodynamic equilibrium. This agrees with the short-lived, low quantum-yield, two-band emission of 1-pyren-2′-yl-2-phenyl-*o*-carborane reported by Lee and Park.^68^

To understand further the influence of solvent polarity on the thermodynamic equilibrium between the excited states, dioxane (2–10%) was added stepwise to a hexane solution of 1 (Fig. 4b). While the LE band does not shift at all upon addition of dioxane, the CT band red shifts because of the stabilization of the CT states by the more polar solvent mixture (increasing dioxane concentration). This makes the LE–CT band gap larger, which slows *k*_−CT_, reduces the population of the LE state, and decreases the LE : CT band ratio in solution with increasing dioxane concentration. With 10% dioxane added, the CT state is stabilized by *ca.* 0.15 eV, as calculated from the 0–0 transition energy in the emission spectra, indicating an increase of the equilibrium constant *K* by a factor of 328.

### Femto- and nano-second transient absorption spectroscopy

**Fig. 5:**
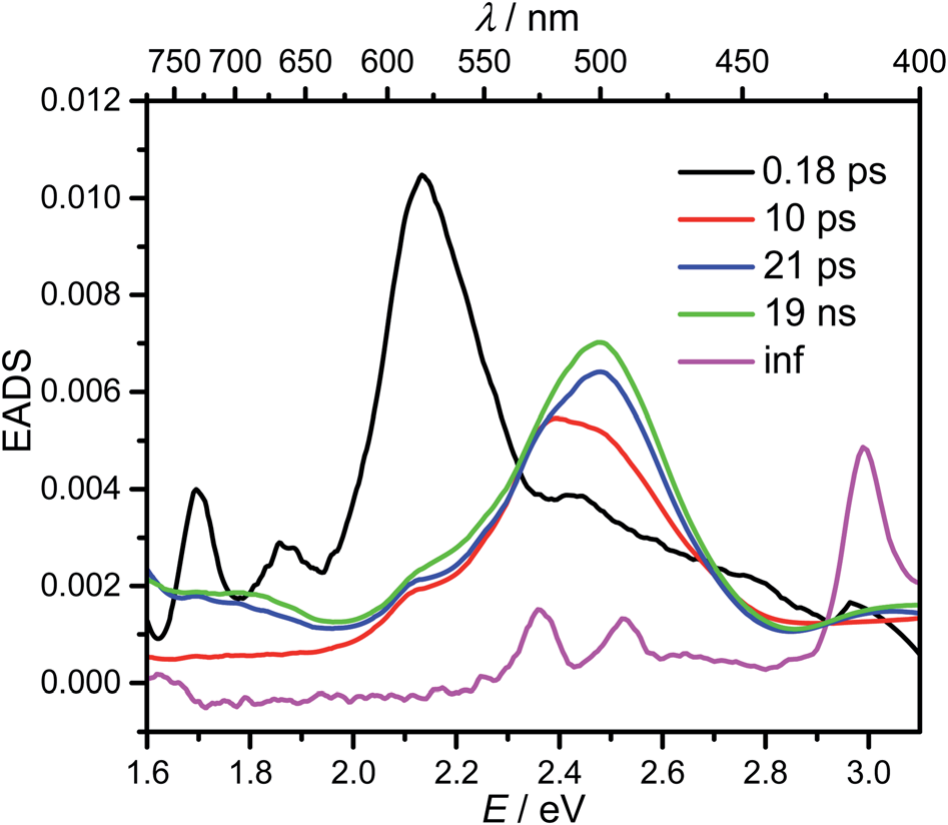
Evolution associated difference spectra (EADS) from a global deconvolution of the transient absorption spectra of 1 in hexane at room temperature, excited at 333 nm (3.72 eV).

To gain more detailed insight into the short time scale kinetics of the photoinduced processes we performed transient absorption spectroscopy with fs time resolution on compound 1 in hexane solution. The sample was pumped at 333 nm (3.72 eV) with a 140 fs pulse generated by an amplified Ti-sapphire oscillator using an optical parametric amplifier. At this wavelength, the 0–0-band of the pyrene S_2_ state is populated. Transient changes of the optical density were probed by a white light pulse (*ca.* 140 fs) generated using a CaF_2_ crystal. The time delay was adjusted by using a stage in double pass retroreflection mode in a combination of linear (20 fs) and logarithmic steps between time zero and 8 ns. The chirp and stray light corrected transient spectra can be found in the ESI (Fig. S7†) and reveal, at first sight, complicated kinetics. Thus, we performed a global deconvolution using GLOTARAN software^69,70^ which yields five evolution associated difference spectra (EADS), assuming a linear model with increasing lifetimes, which are displayed in Fig. 5. In principle, one can also fit the transient map to a branched kinetic model, but this would require knowledge of the specific efficiencies of individual steps and extinction coefficients of the individual species associated difference spectra which are not available in this case. Nevertheless, one can extract considerable semiquantitative information from the EADS.

**Fig. 6:**
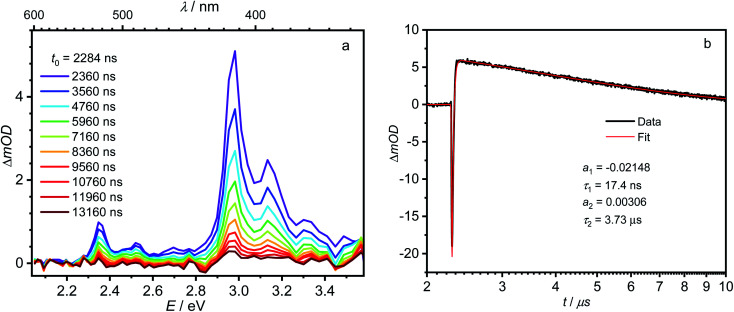
(a) Nanosecond transient absorption spectra of 1 in hexane at room temperature, observed 2360–13 160 ns after excitation at 380 nm (3.26 eV). (b) Time scan and biexponential fit at 414 nm (3.00 eV).

**Fig. 7:**
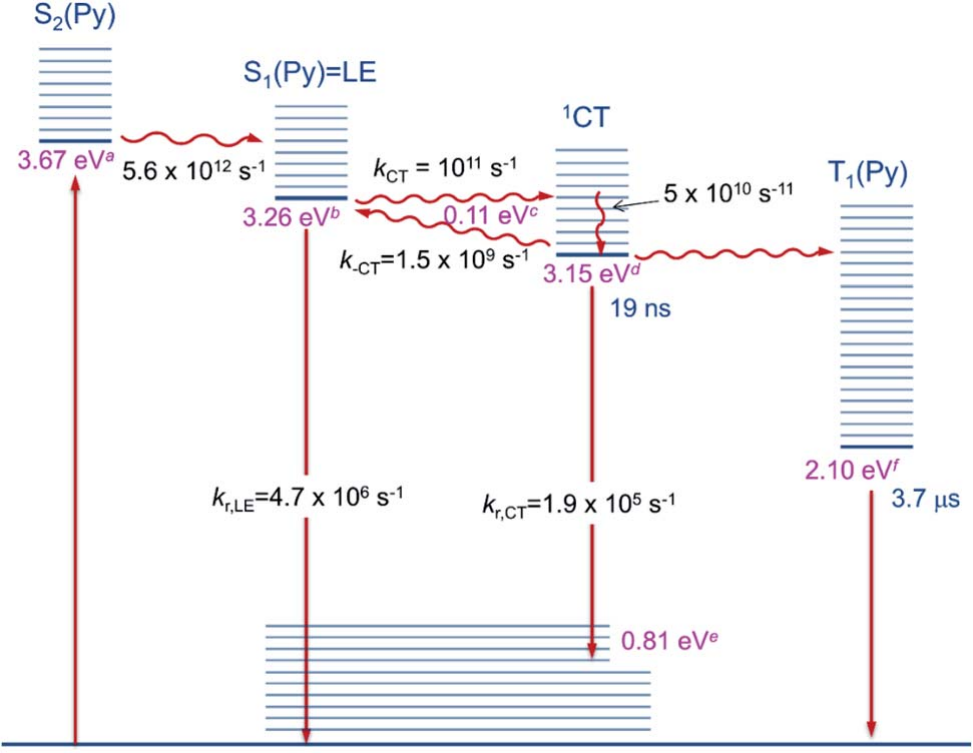
Diagram of the excited state relaxation processes. ^a^From 00-energies of the absorption spectrum; ^b^from 00-energies of the emission spectrum; ^c^from the Stevens–Ban plot; ^d^from b and c; ^e^from d–*E*_max(em)_; ^f^see ref. 74.

The first component (black) with a lifetime of 180 fs is clearly caused by excited state absorption (ESA) of the initially populated pyrene S_2_ state with a prominent peak at *ca.* 580 nm (2.13 eV).^71,72^ This spectrum is followed by an EADS (red) with *τ* = 10 ps which refers to the pyrene S_1_ state (= LE state) with an ESA at *ca.* 500 nm (2.48 eV). The 180 fs thus refers to the internal conversion S_2_ → S_1_ within the pyrene chromophore, in reasonable agreement with values reported for isolated pyrene.^71,72^ The third and fourth EADS (blue and green) possess lifetimes of 21 ps and 19 ns, respectively. They are spectrally very similar, and we assign them to be the hot and cooled singlet CT state. The 21 ps then refers to a vibrational cooling process. We note that this fast vibrational cooling in S_1_(LE) justifies the use of a thermodynamic analysis to characterize the adiabatic LE–CT interconversion. On the other hand, the 19 ns is in excellent agreement with the fluorescence lifetime of 22 ns as measured by TCSPC above, although we have to stress that the 19 ns lifetime is associated with a major error as our delay line covers 8 ns maximum. With care, we thus can assign the 10 ps to the charge transfer process between the LE and the CT state. Here we have to stress that we cannot extract rate constants as this would require the knowledge of the efficiencies of each process. However, the reciprocal lifetime can serve as a rough guide for the rate constant which is *k*_CT_ ≈ 1/10 ps = 10^11^ s^−1^ and with Δ*G* = 0.11 eV using 
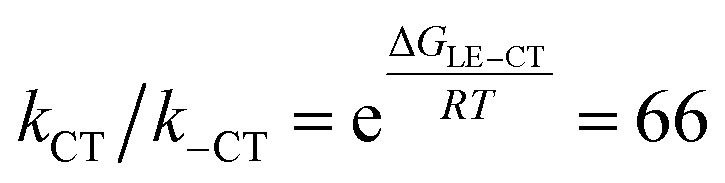
 yields *k*_−CT_ ≈ 1.5 × 10^9^ s^−1^. The global deconvolution also gives an EADS with small amplitude and infinite lifetime, but with very characteristic peaks at 415 nm (2.99 eV), 480 nm (2.58 eV), and 525 nm (2.36 eV). These are caused by a local pyrene triplet state. Indeed, laser flash spectroscopy yields identical transient spectra (see Fig. 6a) in this wavelength region in agreement with literature spectra.^73^ The time trace at 414 nm (2.99 eV) shows, initially, a negative signal corresponding to emission from the LE state and then a rise with *τ*_1_ = 17.4 ns to yield a positive signal corresponding to the triplet ESA which then decays with *τ*_2_ = 3.7 μs. The rise time is in good agreement with the fluorescence lifetime of the LE/CT states and shows that the triplet state is populated *via* either the LE or the CT state. However, as it obviously does not drain the LE–CT equilibrium to a significant extent, intersystem crossing to the triplet state must be slower than any other deactivation process. Taking all information from the steady state and time resolved optical experiments together we can now set up a state diagram as shown in Fig. 7.

### Theoretical studies

Using DFT calculations at the CAM-B3LYP/6-31G(d) level of theory, the geometry optimized ground-state (S_0_) of 1 was found to have a dihedral angle C2–C1–C3–C4 (*ψ*, for the numbering of atoms, see Fig. 1) of 29.8° and a C1–C2 bond length of 1.631 Å. These agree with the solid-state geometries determined by X-ray diffraction (*vide infra*) with *ψ* between 24.2(6)–39.9(6)° and C1–C2 bond lengths between 1.638(6)–1.647(6) Å. A rotational energy profile was constructed for 1 by changing *ψ*, and a very small energy barrier was found for the rotation, between 0 ≤ *ψ* ≤ 90° (see Fig. S8†). The highest energy on this rotational energy surface was calculated to be only 0.015 eV (1.4 kJ mol^−1^) at *ψ* = 90°, indicating that all geometries with 0 ≤ *ψ* ≤ 90° are accessible at room temperature with nearly equal populations.

**Fig. 8:**
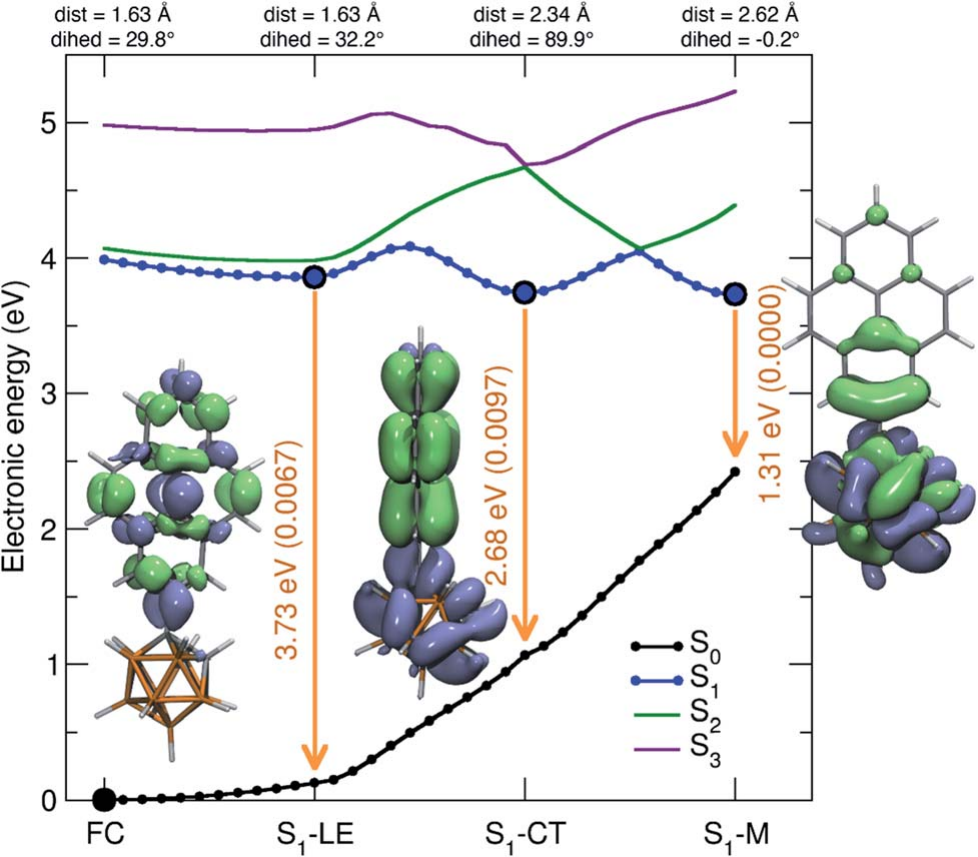
Analysis of the S_1_ potential energy surface of 1. The term “dist” refers to the C1–C2 distance, and “dihed” refers to the dihedral angle between the C1–C2 bond and the aryl plane of pyrene. The different minima of the S_1_ potential energy surface are given by large blue circles (the S_0_ minimum is indicated by a black circle). Linear interpolations in internal coordinate (LIIC) pathways between the different critical points of the first excited-state potential energy surface of 1 are indicated by the blue line. The energies of the other electronic states along the LIIC pathways are given by black (S_0_), green (S_2_), and palatinate (S_3_) lines. Insets provide the molecular geometries of the three S_1_ minima, as well as the corresponding S_0_/S_1_ transition density. For all molecular structures, the C1–C2 bond of the carborane is shown in the plane of the paper.

To shed light on the possible emission processes of 1, the potential energy surface (PES) of its first excited electronic state, S_1_, was studied using linear-response time-dependent density functional theory (LR-TDDFT) with the long-range corrected CAM-B3LYP functional (see Computational details for more information). First, geometry optimizations were conducted on the S_1_ PES of 1 to locate possible minima. In the following, we will focus on three minima, named S_1_–LE (locally excited), S_1_–CT (charge transfer), and S_1_–M (mixed), indicated by large blue circles in Fig. 8. These three minima are close in electronic energy: *E*(S_1_–LE) = 3.86 eV, *E*(S_1_–CT) = 3.74 eV, and *E*(S_1_–M) = 3.73 eV. All electronic energies are given with respect to the optimized ground state geometry, taken as the Franck–Condon (FC) geometry (large black circle in Fig. 8). The molecular geometry of S_1_–LE (left molecular structure in Fig. 8) is close to the optimized ground state geometry. S_1_–CT (central molecular structure in Fig. 8) shows a 90° twist of the pyrenyl moiety with respect to the C1–C2 bond of the carborane. This C1–C2 bond is notably longer in S_1_–CT than in S_1_–LE, with a value of 2.34 Å compared to 1.63 Å, respectively (for reference, the ground-state optimized structure shows a C–C bond length of 1.63 Å), in agreement with that in an *o*-carborane radical anion (2.37/2.39/2.311(3) Å).^75–77^ The bond length is even more stretched when reaching the S_1_–M minimum (right molecular structure in Fig. 8), which has a C–C distance of 2.62 Å. We note that another minimum with S_1_–LE character was found, with a geometry exhibiting a 90° twist of the pyrenyl group. This structure, however, has an almost identical electronic energy and vertical transition energy to that of S_1_–LE.

Transition density plots provide access to the electronic character of the different S_1_ minima by highlighting the changes in electronic density between the excited and the ground electronic states (lime-green in the isosurface plots identify a density depletion upon excitation, and ice-blue a density increase). The transition density plot for the S_1_–LE minimum shows the locally-excited nature of S_1_ at this nuclear configuration (left molecular structure in Fig. 8), as the transition density is essentially localized on the pyrenyl moiety. The transition density for the S_1_–CT minimum shows a significant charge transfer from the pyrenyl moiety to the carborane, while the one obtained for the S_1_–M state is mostly localized on the carborane, with a donating contribution from the closest part of the pyrenyl moiety.

The vertical emission energies for the different S_1_ minima are indicated in Fig. 8 by orange arrows and are summarized here. The largest transition energy is observed for S_1_–LE (3.73 eV, 332 nm), with a small, yet non-zero, oscillator strength of 0.0067. Experience shows that long-range corrected functionals, such as CAM-B3LYP, are likely to give transition energies which are too high for an excited state with a valence character. Hence, the transition energy for S_1_–LE computed with CAM-B3LYP is likely to be overestimated (see also comparison with the emission spectrum below). The vertical emission from S_1_–CT is calculated to occur at 2.68 eV (463 nm), with an oscillator strength of 0.0097. The smaller S_1_/S_0_ gap for this transition, as compared to the one of S_1_–LE, can be explained by the carborane C1–C2 bond stretch observed at this geometry which leads to a destabilization of the ground electronic state (see S_1_ and S_0_ energies at the S_1_–CT geometry in Fig. 8). This destabilization is dramatically amplified when moving towards the S_1_–M geometry, resulting in a vertical transition energy of 1.31 eV (947 nm). The oscillator strength is zero at this particular geometry, and this transition was not detected in the emission spectra. In summary, while the different S_1_ minima located have very similar electronic energies, they show a broad range of vertical transition energies due to the underlying destabilization of the ground electronic state.

Up to this point, minima on the S_1_ potential energy surface were characterized in terms of electronic energy, electronic character, and vertical transition energy. We now turn to the possible photophysical processes of 1 on the S_1_ surface. For this purpose, we calculated pathways connecting the three minima and the Franck–Condon point discussed previously. We start by considering pathways produced by linear interpolations in internal coordinates (LIIC). The idea behind exploring LIIC pathways is to determine the most straightforward path from geometry A to geometry B by interpolating a series of geometries in between, using internal coordinates (not Cartesian). Importantly, no reoptimization is performed along these pathways, implying that LIIC pathways should *not* be seen as minimum energy paths, *per se*, and the barriers observed in LIICs are possibly higher than the actual barriers one would obtain by searching for proper transition states. LIIC pathways offer a preliminary picture of the possible photophysical and photochemical processes that a molecule can undergo.

In the S_1_ state, the molecule can relax from the FC region towards the S_1_–LE minimum without encountering any barrier, based on the LIIC pathway (Fig. 8). The S_1_–LE minimum lies 0.13 eV below the FC point. From the S_1_–LE minimum, the LIIC pathway indicates that the molecule needs to overcome a barrier of 0.23 eV to reach the S_1_–CT minimum (0.34 eV in the other direction). The last LIIC pathway connects S_1_–CT to S_1_–M, and shows a barrier of 0.31 eV. Interestingly, the change of electronic character can be clearly identified along this pathway; the energy gap between S_1_ and S_2_ becomes nearly zero halfway through the path (see S_1_/S_2_ in Fig. 8).

**Fig. 9:**
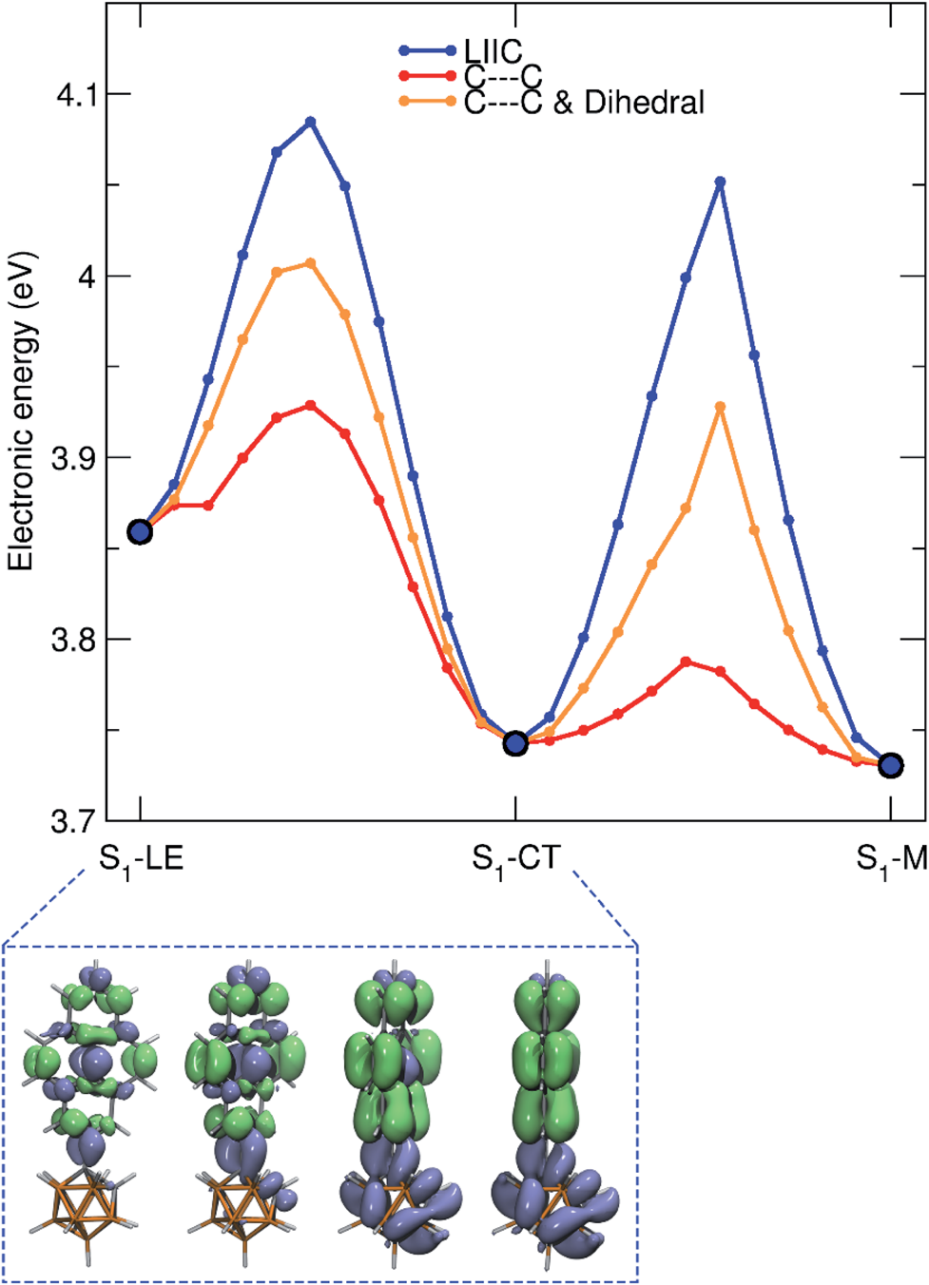
LIIC pathways computed between the S_1_–LE, S_1_–CT, and S_1_–M minima (blue line) and relaxed scans on the S_1_ electronic states. Relaxed scans were performed along different coordinates: the C1–C2 bond length of the carborane alone (C1–C2) or combined with the dihedral angle between the pyrenyl moiety and the C1–C2 bond of the carborane (C1–C2 & dihedral). The connected minima define the initial and final values for the scanned coordinates. The lower panel shows the S_0_/S_1_ transition density along the LIIC pathway between S_1_–LE and S_1_–CT.

As mentioned earlier, the LIIC pathways do not imply a relaxation of the geometry along the path, and an LIIC barrier should be seen as an upper limit to a true barrier. Complementary relaxed scans, obtained by minimizing the S_1_ geometry subject to a fixed C1–C2 bond length in the carborane or to a fixed C–C bond length combined with a fixed dihedral angle of the pyrenyl moiety with respect to the C1–C2 axis, were performed to refine the values of the barriers (Fig. 9). All relaxed scans show a smaller barrier for the transition from S_1_–LE to S_1_–CT with a value as low as 0.07 eV for a smooth scan performed along the C1–C2 bond. For the S_1_–CT to S_1_–M pathway, a similar behaviour is observed with a barrier computed at 0.05 eV. Therefore, LR-TDDFT/CAM-B3LYP indicates that the molecule can possibly visit all three S_1_ minima following photoexcitation, smoothly changing its electronic character from LE to CT (as depicted in the lower panel of Fig. 9) and then from CT to M.

**Fig. 10:**
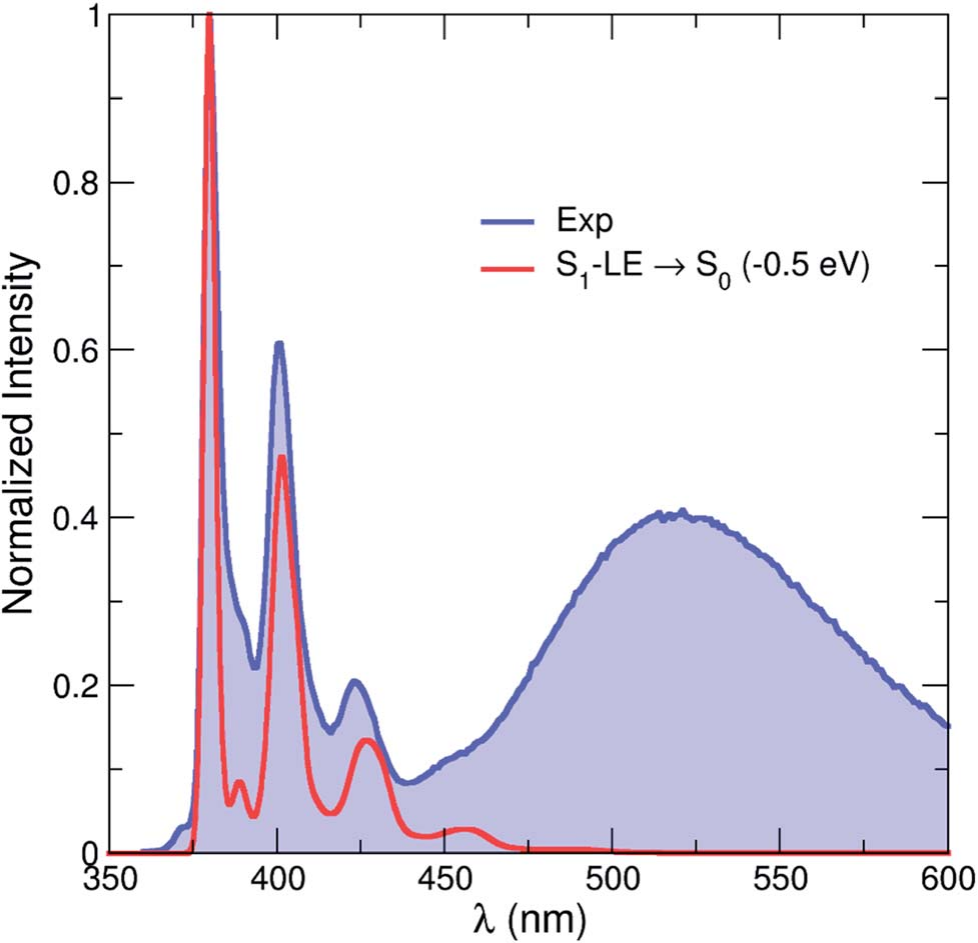
Comparison between the experimental (blue line and area) emission spectrum in hexane at room temperature and that calculated by CAM-B3LYP (red line, rigidly shifted by −0.5 eV). The theoretical emission spectrum only contains the contribution from the S_1_–LE state.

A theoretical emission spectrum, including the role of vibronic progressions, can be computed from the S_1_–LE geometry described above. This is achieved by invoking an harmonic approximation for the normal modes of the molecule (at the S_0_ and the S_1_–LE optimized geometries) and by computing Franck–Condon and Herzberg–Teller terms.^78^ The resulting emission spectrum is shown in Fig. 10 (and S9†) and its vibronic progression is in close agreement with the one observed experimentally for the high-energy (LE) part of the emission spectrum. This result constitutes a strong validation of the S_1_–LE structure obtained with CAM-B3LYP. (We note that the theoretical spectrum was rigidly shifted by −0.5 eV due to the aforementioned tendency of CAM-B3LYP to overestimate valence transition energies.) Unfortunately, the important difference in geometries between the S_0_ optimized geometry and the S_1_–CT one hampers the use of the harmonic approximation and prevents the calculation of the corresponding low-energy emission band using this strategy.

In summary, these calculations indicate that emission could take place from different minima on the S_1_ potential energy surface of 1. LIIC pathways at the LR-TDDFT/CAM-B3LYP level of theory suggest that the molecule could visit the three minima studied here, with S_1_–LE and S_1_–CT being favoured candidates for the dual emission (based on their experimentally observed emission properties). The calculations presented here do not account for solvent effects. The energies of the minima, as well as the LIIC pathways, are likely to be altered by the inclusion of solvent effects, in particular the minima showing a CT character. In addition, the stretch of the C1–C2 bond of the carborane can potentially challenge the accuracy of LR-TDDFT.

### Solid-state structure

The solid-state structure (Fig. 1) was determined by single-crystal X-ray diffraction at 100 K. The crystal structure of 1-(pyren-2′-yl)-*o*-carborane is composed of π-stacked molecules with stacks running parallel to the *a* axis. Two stacks can be distinguished, each of which consists of two non-symmetry-equivalent molecules alternating within the stack. In one of the stacks the molecules are rotated around the stacking axis (*a* axis) by 142.3(3)°, calculated from the angle between the C1–C3 bonds which connect the pyrene to the carborane moieties (Fig. 1 and S7†). In this stack the pyrene planes of the molecules, which are defined by their normals, are tilted 16.0(2)° and 15.7(2)° with respect to the stacking axis (Fig. S10†). They are π-stacked with mean intermolecular distances of 3.446(1) Å and 3.420(5) Å alternating along the stacking direction. The other stack exhibits a smaller rotation of the molecules with the C1–C3 bonds of the molecules forming an angle of 44.5(3)° (Fig. S11†). The tilt of the pyrenyl planes to the stacking axis is smaller being 6.5(2)° and 5.6(2)°, respectively (Fig. S10†). Hence, the molecules are oriented nearly perpendicular to the stacking axis. In turn, the mean intermolecular distances within this stack are a little longer, being 3.587(1) Å and 3.511(8) Å, alternating along the stacking axis. Within the crystal structure, stacks are related *via* inversion symmetry. The C1–C2 bonds within the carborane cluster are tilted out of the pyrenyl plane in all four non-symmetry-equivalent molecules (Fig. 1). The angles between the C1–C2 bonds and the pyrenyl planes are 36.1(2)°, 21.3(2)°, 26.6(2)°, and 30.7(2)° for molecules 1, 2, 3, and 4, respectively. The C2–C1–C3–C4 (or C2–C1–C3–C16 in molecules 1 and 4, respectively) dihedral angles are 39.9(6)°, −24.2(6)°, 30.6(6)°, and −33.5(6)° for molecules 1, 2, 3, and 4, respectively (Fig. 1).

### Solid-state emission

A crystalline sample of 1 shows only a broad emission band (Fig. S12†) with a maximum at 2.65 eV (468 nm) with a relatively short lifetime (*τ* = 3.4 ns) and higher quantum yield (*Φ* = 24%). This is probably fluorescence from a pyrene excimer due to the π–π stacking of the pyrene units (*vide supra*). This is different from 1-(pyren-1'-yl)-*o*-carborane (2),^47^ which shows temperature-dependent dual-fluorescence in the solid state.^79^ The LE : CT band ratio of 2 in the solid state is larger at lower temperature, probably because the rotation of the carborane is more restricted at lower temperature, indicating a kinetically-controlled process.

## Conclusions

Taking 1-(pyren-2′-yl)-*o*-carborane (1) as a model, we studied the photoinduced CT process and the thermodynamic equilibrium between the CT state and the thermally activated LE state, by combining steady state, time-resolved, and temperature-dependent fluorescence spectroscopy, fs- and ns-transient absorption, and DFT and LR-TDDFT calculations.

The absorption spectra of 1 suggest that only the LE state can be accessed by excitation, and the CT state is populated from the LE state. Conversion of the LE state to the CT state occurs in solution, but is impeded in a frozen matrix or the crystalline state. The compound shows only a pyrene-like emission band in a frozen matrix, and an excimer emission in the crystalline state.

Time-resolved fluorescence spectroscopy of 1 in hexane indicates that the LE and CT states have the same lifetimes and are in thermodynamic equilibrium in hexane at room temperature. The lifetimes of the two bands became longer, but maintained the same ratio upon cooling the solution to 188 K from room temperature, suggesting that interconversion between the two states is rapid in this temperature range. A Stevens–Ban plot reveals that the LE state lies 0.11 eV above the CT state. Decreasing the temperature or increasing the polarity of the solvent enhances the population of the CT state, thus decreasing the LE : CT band ratio.

Femtosecond transient absorption spectra reveal that, after local excitation, the excited molecule converts to the CT state in *ca.* 10 ps, confirming that the internal conversion rate is much faster than the fluorescence decay rate and any other excited-state process, indicating a very small internal conversion barrier. They also confirmed the CT nature, rather than a full “electron-transfer” for the “CT” state.

Calculations at the LR-TDDFT/CAM-B3LYP level of theory indicate the presence of at least three different key minima on the S_1_ potential energy surface corresponding to an LE, a CT, and a mixed state. The energetics of the LE and CT states are in close agreement with experimental evidence, and an emission spectrum computed from the LE minimum accurately reproduces the shape of the experimental LE spectrum. An LIIC pathway connecting the different minima indicates that internal conversions between the different minima are possible at room temperature. In particular, the distortion upon going from S_1_–LE to S_1_–CT combines a twist of the pyrene group with an elongation of the C–C bond of the carborane.

The results reported in this paper suggest caution when determining whether the excited states of a dual-emissive compound are in thermodynamic equilibrium or, for example, defining whether an emission is due to TADF. Thus, a one-band, single-lifetime emission could potentially arise from two or more excited states, including a T_1_ state.

## Experimental

### General manipulations and synthetic techniques

The compound 2-ethynylpyrene was synthesized according to a literature procedure.^62,63^ Toluene, acetonitrile, chloroform, tetrahydrofuran, and hexane were dried using an Innovative Technology Inc. solvent purification system (SPS) and degassed before use. Dioxane was distilled over sodium and stored at 4 °C (solid state) before use. NMR spectra were recorded in CDCl_3_ solution on a Bruker Advance 500 NMR spectrometer (^1^H, 500 MHz; ^13^C, 125 MHz; ^11^B, 160 MHz). ^1^H NMR spectra are referenced to TMS *via* the signal of the residual protonated solvent. ^13^C{^1^H} NMR spectra are referenced to TMS *via* the ^13^C resonance of the deuterated solvent. ^11^B NMR spectra are referenced to external BF_3_·Et_2_O. Mass spectra were obtained using an Agilent Technologies GC-MS system (GC 7890A, EI MS 5975C).

#### Synthesis of 1

The compounds 2-ethynylpyrene (0.58 g, 2.6 mmol) and B_10_H_14_ (0.32 g, 2.6 mmol) were placed in an argon-filled Schlenk tube. Dry, degassed toluene (10 mL) and MeCN (5 mL) were added under an argon atmosphere. The Schlenk tube was attached to an oil bubbler, and the reaction mixture was stirred for 3 days at 110 °C. After removing solvent *in vacuo*, the crude product was purified by column chromatography on silica using dichloromethane/hexane 1 : 9 as eluent, followed by recrystallization from hot hexane, to give 1 as thin yellowish needle-like crystals (0.25 g, 28%). ^1^H NMR (500 MHz, CDCl_3_): *δ* = 8.25 (s, 2H), 8.23 (d, *J* = 8 Hz, 2H), 8.14 (d, *J* = 9 Hz, 2H), 8.08–8.03 (m, 3H), 4.26 (s, 1H), 2.83 (s, 2H), 2.76 (s, 2H), 2.54 (s, 1H), 2.43 (s, 2H), 2.39 (s, 2H), 3.20–2.00 (br, 1H) ppm. ^13^C{^1^H} NMR (126 MHz, acetone-d_6_): *δ* = 131.21, 132.17, 132.02, 129.74, 127.99, 127.96, 126.78, 125.29, 124.52, 124.28, 78.55, 62.13 ppm. ^11^B{^1^H} NMR (160 MHz, CDCl_3_): *δ* = −1.9, −4.2, −8.7, −10.4, −10.8, −12.6 ppm. MS (EI^+^): *m*/*z* 345 [M^+^]. Elem. anal. calcd for C_18_H_20_B_10_: C 62.76, H 5.85; found: 62.40, 6.01. Molar extinction coefficients (in hexane, room temperature): *ε* (379 nm) = 400 M^−1^ cm^−1^, *ε* (338 nm) = 53 900 M^−1^ cm^−1^, *ε* (279 nm) = 35 200 M^−1^ cm^−1^, *ε* (253 nm) = 82 400 M^−1^ cm^−1^.

### Photophysical measurements

The concentrations of 1 used in all photophysical measurements were *ca.* 5 × 10^−6^ M in hexane while those used for the emission spectra have a maximum absorbance less than 0.2 to avoid re-absorption. Absorption spectra were recorded on an Agilent 8453 diode-array UV-Vis spectrophotometer. The emission and excitation spectra were recorded using an Edinburgh Instruments FLSP 920 spectrometer equipped with double monochromators for both excitation and emission, operating in right angle geometry mode. The fluorescence quantum yields were measured using a calibrated integrating sphere (150 mm inner diameter). Lifetime measurements were conducted using the time-correlated single-photon counting method (TCSPC) on the FLSP 920 spectrometer equipped with a high-speed photomultiplier tube positioned after a single emission monochromator. Decays were recorded to 10 000 counts in the peak channel with a record length of at least 1000 channels. The quality of all decay fits was judged to be satisfactory, based on the calculated values of the reduced *χ*^2^ and Durbin–Watson parameters and visual inspection of the weighted and autocorrelated residuals. The lifetimes of 1 in polar solvents were not recorded as the emissions were too weak. All absorption and emission spectra were recorded in standard quartz cuvettes (1 cm × 1 cm) under argon.

### Femtosecond transient absorption spectroscopy

All experiments were performed in quartz cuvettes with an optical path length of 2 mm equipped with a micro stirrer at room temperature. All samples were dissolved in hexane, filtered and degassed. The optical density was adjusted to *ca.* 0.2 at the corresponding excitation wavenumber. The transient absorption spectra were performed with a Newport-Spectra-Physics Solstice one-box amplified ultrafast Ti : Sapphire laser system with a fundamental wavenumber of 800 nm, a pulse length of 100 fs and a repetition rate of 1 kHz.

The output beam from the Solstice amplifier was split into two parts. One part was focussed onto a vertically oscillating CaF_2_ crystal to produce a white light continuum between 850 nm and 350 nm. The resulting beam, which was polarized horizontally, was used as the probe pulse. The second pulse was used to pump an optical parametric amplifier (TOPAS-C) from Light Conversion to generate the pump pulse with a pulse length of 140 fs at the 333 nm excitation wavelength. By using a wire grid (Moxtek) the polarization axis of the pump pulse was set to the magic angle relative to the probe pulse. The pump pulse (50 nJ, *Ø ca.* 0.4 mm) and the probe pulse (*Ø ca.* 0.1 mm) met at an angle of 6° vertically in the cuvette. The probe pulse is measured by means of a CMOS sensor (Ultrafast Systems, Helios). To compensate for white light intensity fluctuations, a reference beam was split off and detected with an identical spectrograph. Every second probe pulse was blocked by a mechanical chopper (500 Hz) to measure the ratio of *I* and *I*_0_.

The computer-controlled stage (retro reflector in double pass setup) set the time difference between pump and probe pulse in 20 fs intervals from 0 fs to 4 ps and 4 ps to 8 ns in logarithmic steps with a maximum step width of 200 ps.

Before data analysis, the raw transient data were corrected for stray light and white light dispersion (chirp). The chirp was corrected by fitting a polynomial to the cross phase modulation signal of the pure solvent under otherwise experimental conditions. The IRF was 280 fs. The evolution associated difference spectra (EADS) were obtained from the corrected data by a global analysis using GLOTARAN software.

### Nanosecond transient absorption spectroscopy

All measurements were carried out in a 1 cm quartz cell (Starna) in oxygen-free hexane solutions at room temperature using an Edinburgh Instruments LP 920 laser flash spectrometer. The sample was excited with *ca.* 5 ns laser pulses from the 380 nm output of an EKS-PLA NT 342A Nd:YAG shifted to the desired wavenumber by an OPO BBO II optical parametric oscillator. The excitation pulse had an energy of *ca.* 1.2 mJ.

White light was provided by a pulsed Xe flash lamp. All measurements were carried out with activated fluorescence correction implemented in the L900 software and the time range was chosen such that the decay profile was completely back to zero. For all measurements, a long pass (LP) filter (>400 nm) was placed in front of the detector slit to avoid signals of higher order. The instrument response (*ca.* 7 ns) of the set-up was determined by measuring the scattered excitation pulse using a LUDOX AS-30 colloidal silica suspension in water.

### Computational details

All calculations employed density functional theory (DFT) and linear-response time-dependent density functional theory (LR-TDDFT).^80–82^ The long-range corrected exchange–correlation functional CAM-B3LYP^83^ was used together with a 6-31G(d) basis set for all the atoms. LR-TDDFT employed the adiabatic approximation. Ab Initio Molecular Dynamics (LR-TDDFT/CAM-B3LYP/6-31G(d), employing the Tamm–Dancoff approximation and a time step of 0.5 fs) was performed on the S_1_ potential energy surface of 1 to extend the search for potential minima using the GPU-accelerated software TeraChem.^84,85^ Geometry optimizations were conducted *in vacuo* and the nature of all stationary points located was verified by harmonic vibrational frequency calculations. The emission spectrum from S_1_–LE used a combined Herzberg–Teller Franck–Condon method.^86^ All static calculations were carried out with the Gaussian 09 package (revision D),^87^ and molecular representations were produced with VMD version 1.9.2.^88^

### Crystal structure determination

The crystal structure determination of 1-(pyren-2′-yl)-*o*-carborane was performed in two steps. Single crystal growth proved to be difficult, resulting in small and thin needles which scattered very weakly. Hence, single-crystal synchrotron X-ray diffraction data were collected initially (Fig. S13, see ESI† for Experimental details). As the synchrotron data did not reveal satisfactory structure refinements, new effort was put into crystal growth using alternative techniques (see section on single-crystal growth). The majority of the crystals were much longer, but still thin needles, which were often bent. They diffracted better on the home laboratory diffractometers than the crystals from the first growth procedure, but data were pointing towards crystal imperfections resulting in diffuse streaks and multiple domains. A minority of crystals with a different, more compact needle-like habit formed rose-like intergrowths. Twinned single crystal needles could be broken from these intergrowths which showed sufficiently good and separated diffraction. The diffraction data of one of the crystals allowed us to solve the crystal structure and refine it to satisfactory residual values.

#### Single-crystal growth

The thermal stability of the pyrene-carborane compound allows for sublimation growth of bulk single crystals by two different routes. In a first attempt, we employed the Lipsett growth technique which benefits from the small amount of material (∼5 mg) necessary as well as from the fast growth of high-quality samples. The Lipsett apparatus consists of a closed growth tube cantered in a vertical oven.^89^ The compound was located at the bottom of the tube and the top was closed by a cold finger made of either glass or metal. The tube can be operated under high-vacuum or at low pressure. In the present case, we sublimed about 10 mg of pyrenyl-carborane, pre-purified by recrystallization, at a temperature of 160 °C and a pressure of *ca.* 300 mbar. The growth proceeded within 4 h and yielded needle-like crystals of *ca.* 5 to 10 mm in length and 50 μm in diameter. However, subsequent X-ray diffraction analysis showed that the quality of the individual crystallites was insufficient to achieve a sufficiently good diffraction pattern for structure determination. Therefore, in an alternative approach, we applied horizontal vapour phase deposition in a temperature gradient under inert gas flow.^90^ For this purpose, the purified material was located in a horizontal oven streamed by N_2_ (purity 6N) at a rate of 30 sccm. Evaporating 23 mg of the pyrenyl-carborane compound at a temperature of 210 °C, the material was transported by the inert gas stream to the colder zone of the oven where it recondensed and formed elongated, needle-like crystals. Overall, the gradient sublimation procedure was carried out for 65 h, yielding crystallites comparable in dimensions to those prepared by Lipsett growth. In contrast, however, X-ray structural analysis revealed much better-defined diffraction patterns due to the improved crystal quality which, finally, enabled the determination of the pyrenyl-carborane unit cell parameters and an improved structure solution from the gradient-sublimation grown crystals.

#### In-house single-crystal X-ray diffraction

One gradient-sublimation grown crystal suitable for single-crystal X-ray diffraction was selected, coated in perfluoropolyether oil, and mounted on a MiTeGen sample holder. Diffraction data were collected on a Bruker D8 Quest diffractometer with a CMOS area detector and multi-layer mirror monochromated Mo-K_α_ radiation. The crystals were cooled using a Bruker Kryoflex II low-temperature device. Data were collected at 100 K. The images were processed and corrected for Lorentz-polarization effects and absorption as implemented in the Bruker software packages. Although two angles of the unit cell are close to 90°, the crystal structure could not be solved with monoclinic symmetry. The structure was solved in space group *P*1̄ using the intrinsic phasing method (SHELXT)^91^ and Fourier expansion technique. The crystal structure model was the same as obtained from the synchrotron data, although of better quality. All non-hydrogen atoms were refined in anisotropic approximation, with hydrogen atoms ‘riding’ in idealized positions, by full-matrix least squares against *F*^2^ of all data, using SHELXL^92^ software. The crystal was a non-merohedral twin with domains rotated by 179.9° around real axis [0.047 1.000 0.254] or reciprocal axis (0 1 0). Only reflections from the larger domain were used in the refinements. In addition, the structure was refined as a twin, applying the twin matrix (1 0 0, 0 −1 0, 0 0 −1). The twin component was refined to 5.1%. A first impression from the structure solution and electron densities suggested ordered carbon positions. Hence, all boron positions surrounding the carbon position of the carborane clusters, to which the pyrene moieties are attached, were systematically checked for the second carbon position. The comparison of the residual values obtained from each refinement confirmed the reliable determination of the second carbon positions in the carborane clusters. The final residual values converged to *R*_1_ = 0.1028 for reflections with *F*_o_ > 4*σ*(*F*_o_) and w*R*^2^ = 0.2695 for all reflections. This was the best result obtained for crystals of this compound. The still relatively high residual values can be attributed to twinning, growth imperfections, and domains which result in slight diffuse scattering. However, in this crystal, Bragg reflections of twin components can be resolved. They are not overlapping along the reciprocal *b⃑*∗ direction in every row with odd *l* indices while overlapping in rows with even *l* indices (Fig. S14†). Nevertheless, the twin component extraction can be problematic, being responsible for the higher residual value. Furthermore, the occurrence of additional twin laws was found by PLATON software^93^ and the separation of intensities into different twin laws and components can also reduce the reliability of intensities, being responsible for the higher residual values. Diamond software^94^ was used for graphical representation. Crystal data and experimental details are listed in Table S1; full structural information has been deposited with the Cambridge Crystallographic Data Centre. CCDC-1863611.†

## Note added in proof

A referee suggested adding the following reference to a review on “Solid-State Luminescent *o*-Carboranes”,^95^ and we also note that a paper on “Dual-Emission Character of Aryl-Modified *o*-Carboranes” from the same group appeared while our paper was being refereed.^96^

## Data availability

Additional data and photophysical spectra, and crystallographic data, NMR spectra, and Cartesian coordinates for calculations can be found in the ESI.†

## Author contributions

L. J. and M. Fest, H. H. A. M. carried out the synthesis and photophysical studies; J. N., B. F. E. C., M. A. F., and D. J. T. carried out the theoretical studies; S. H. carried out the single-crystal growth by sublimation, supervised by J. P.; A. F. carried out the X-ray crystallographic studies; S. C., A. S., and M. H. carried out the transient absorption spectroscopic studies, supervised by C. L.; T. B. M., L. J., C. L., and M. Finze supervised the overall project; all authors were involved in the preparation of the manuscript.

## Conflicts of interest

There are no conflicts to declare.

## Supplementary Material

SC-013-D1SC06867A-s001

SC-013-D1SC06867A-s002
