# Supplementary material for: Thermodynamic equilibrium between locally excited and charge-transfer states through thermally activated charge transfer in 1-(pyren-2′-yl)-o-carborane
Source: Chem Sci. 2022 Apr 19;13(18):5205–19. doi: 10.1039/d1sc06867a (PMC9093154; doi:10.1039/d1sc06867a)
Supplement: SC-013-D1SC06867A-s001 [file SC-013-D1SC06867A-s001.pdf]

Electronic Supplementary Material (ESI) for Chemical Science.
This journal is © The Royal Society of Chemistry 2022

Supporting Information

Thermodynamic Equilibrium Between Locally Excited and Charge-Transfer States through Thermally Activated Charge Transfer in 1-(Pyren-2′-yl)-*o*-carborane

Lei Ji,[a,b*] Stefan Riese,[c] Alexander Schmiedel,[c] Marco Holzapfel,[c] Maximillian Fest,[b] Jörn Nitsch,[b] Basile F. E. Curchod,[d,*] Alexandra Friedrich,[b] Lin Wu,[a] Hamad H. Al Mamari,[b,e] Sebastian Hammer,[f] Jens Pflaum,[f] Mark A. Fox,[d] David J. Tozer,[d] Maik Finze,[b] Christoph Lambert,[c,*] Todd B. Marder[b,*]

[a] Frontiers Science Center for Flexible Electronics, Xi'an Institute of Flexible Electronics (IFE), Northwestern Polytechnical University, 127 West Youyi Road, Xi'an, Shaanxi

[b] Institut für Anorganische Chemie and Institute for Sustainable Chemistry & Catalysis with Boron, Julius-Maximilians-Universität Würzburg, Am Hubland, 97074 Würzburg, Germany

[c] Institut für Organische Chemie, Julius-Maximilians-Universität Würzburg, Am Hubland, 97074 Würzburg, Germany

[d] Department of Chemistry, University of Durham, South Road, Durham, DH1 3LE, U.K.

[e] Department of Chemistry, College of Science, Sultan Qaboos University, PO Box 36, Al Khoudh 123, Muscat, Sultanate of Oman

[f] Experimentelle Physik VI, Julius-Maximilians-Universität Würzburg, Am Hubland, 97074 Würzburg, Germany

Table of Contents

Photophysics in a glass and in solution........2

Theoretical studies........5

Single-crystal X-ray diffraction and solid-state emission........8

NMR Spectra........13

Cartesian coordinates of all optimized states........16

References........18

Photophysics in a glass and in solution

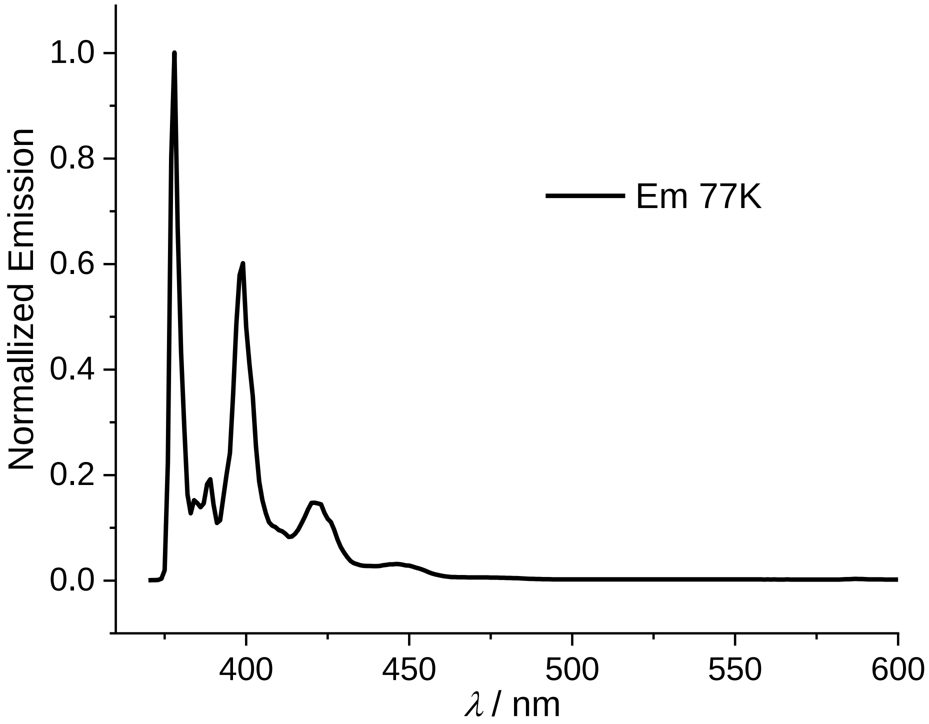

Figure S1. Emission spectrum of compound **1** in a methylcyclohexane glass at 77 K.

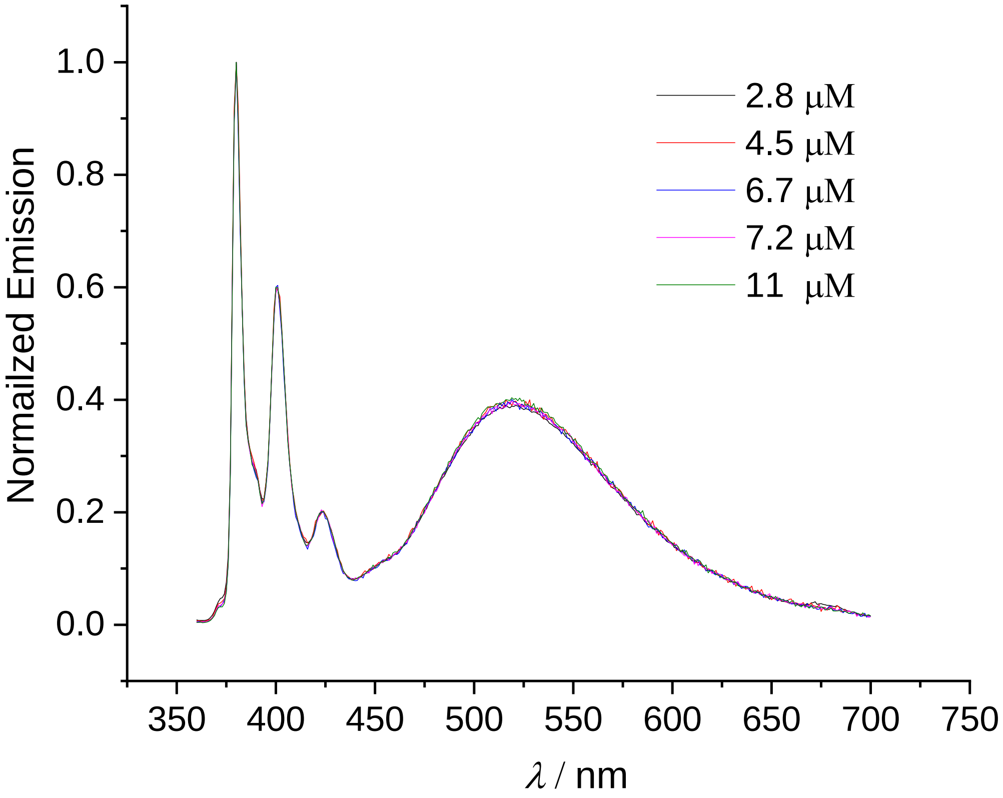

Figure S2. Normalized emission spectra of compound **1** in hexane at different concentrations.

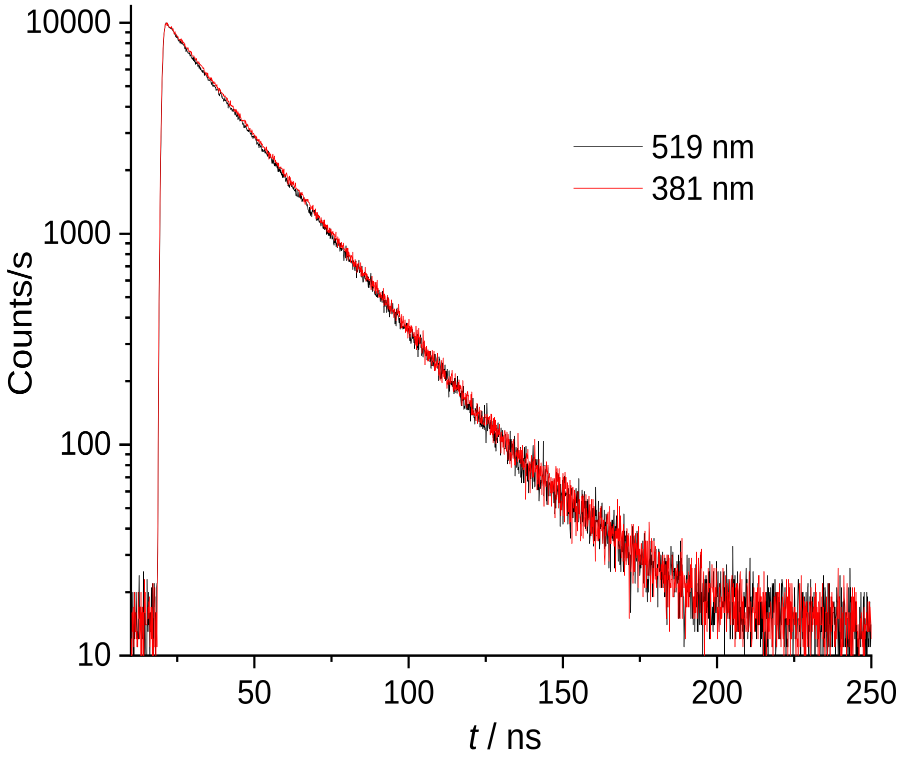

Figure S3. Emission decays at 381 nm and 519 nm after excitation of **1** in degassed hexane (ex @ 338 nm).

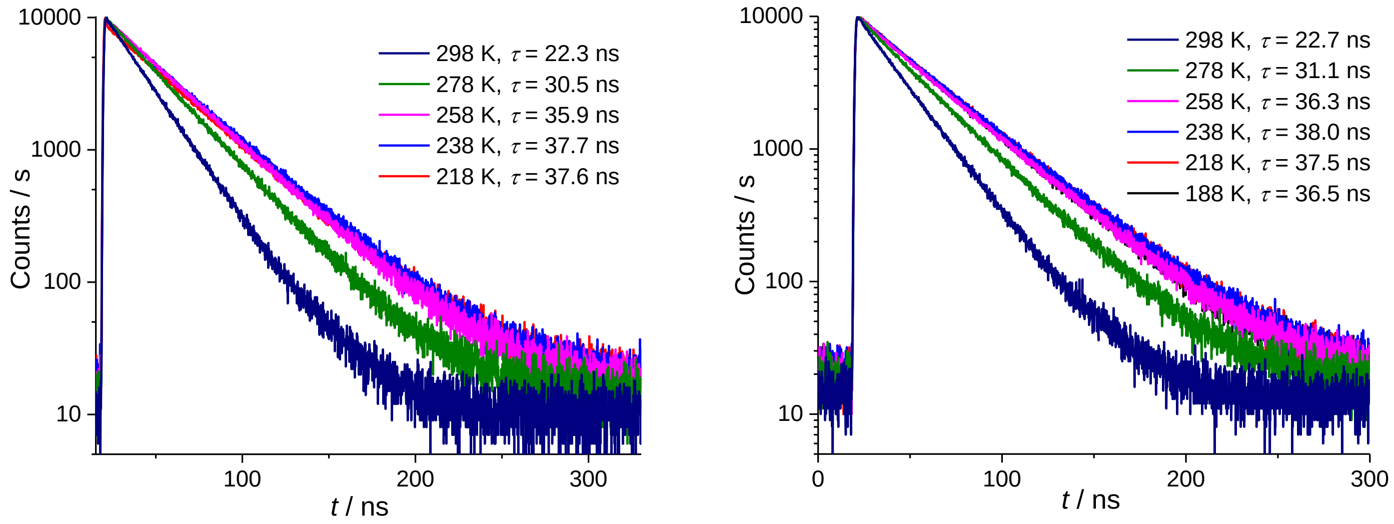

Figure S4: Decays of the LE band (left) and CT band (right) in hexane at different temperatures. At temperatures lower than 218 K, the LE band became so weak and the viscosity of hexane became so large, that scattered light affects the measurements of the LE band, and a fast decay (τ < 1 ns), which is shorter than the instrument response function (1.34 ns full-width-at-half-maximum), was found. However, the lifetime of the longer decay is still the same as that of the CT band.

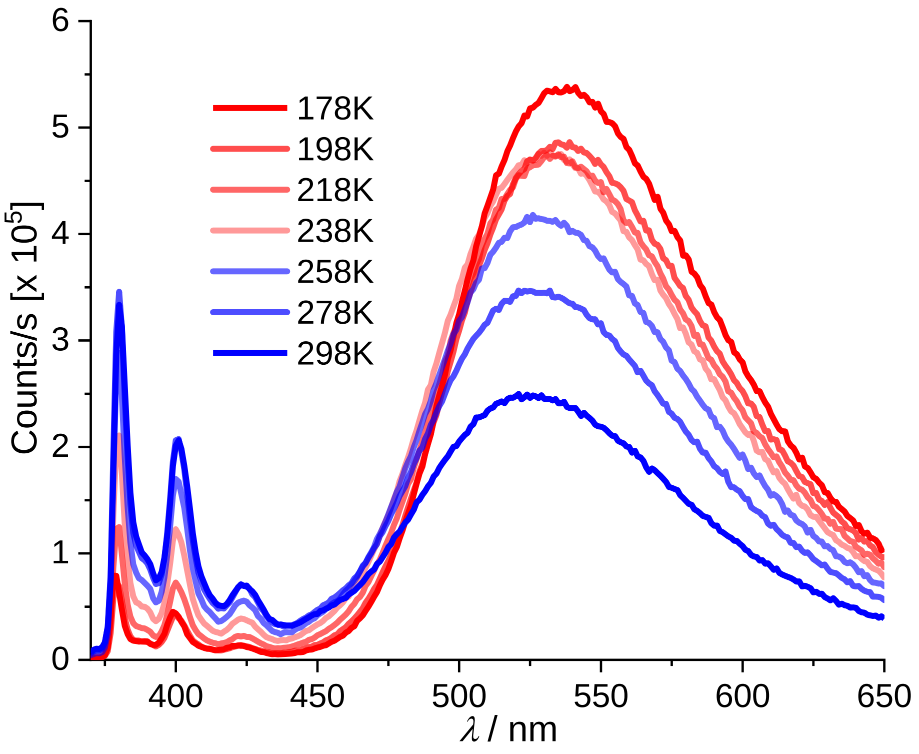

Figure S5. Temperature-dependent emission spectra of **1** in methylcyclohexane.

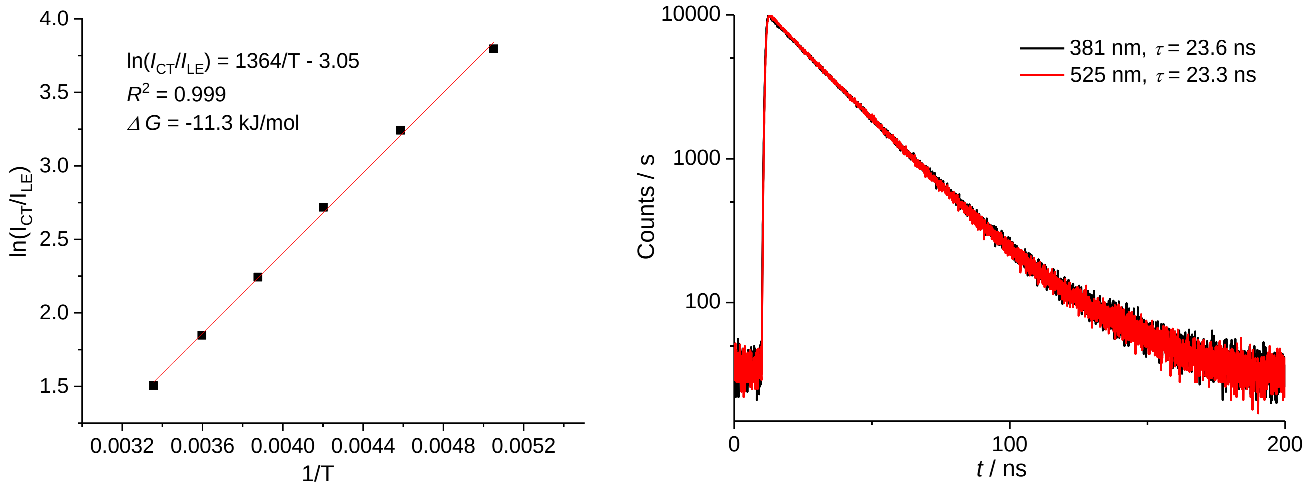

Figure S6. Stevens-Ban plot with linear least-square fit from variable temperature emission spectra in methylcyclohexane (left) and fluorescence lifetimes of compound **1** in methylcyclohexane at 298 K (ex @ 338 nm, right).

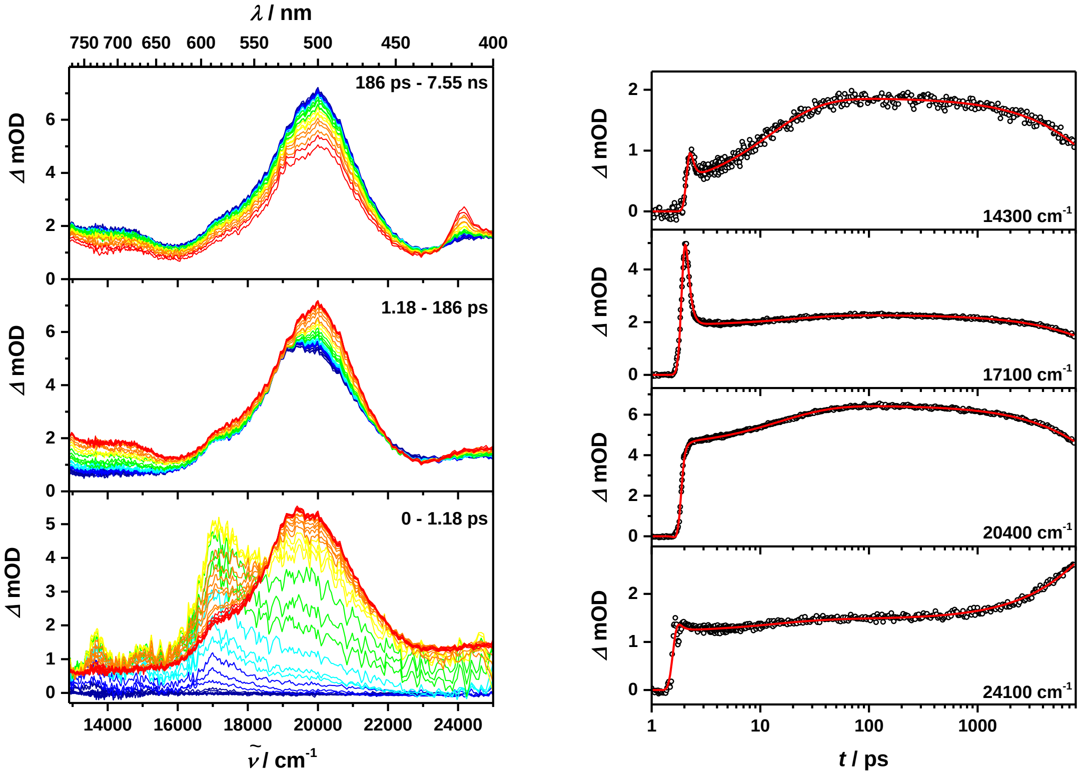

Figure S7. fs-Transient absorption spectra for selected time ranges and time scans at selected wavenumbers with global fit (red line) of **1** at 333 nm excitation in hexane at r.t.

Theoretical studies

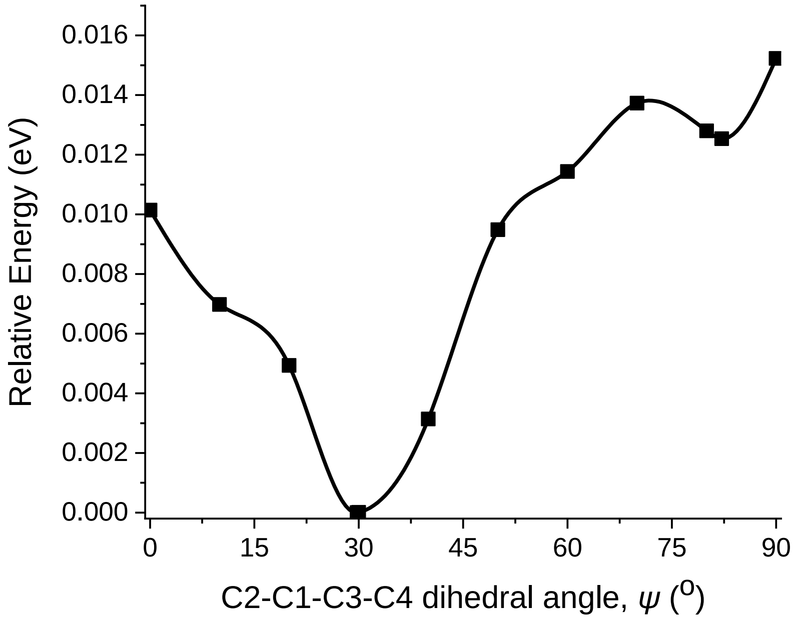

Figure S8. Rotational energy profile for **1** at the CAM-B3LYP/6-31G(d) level.

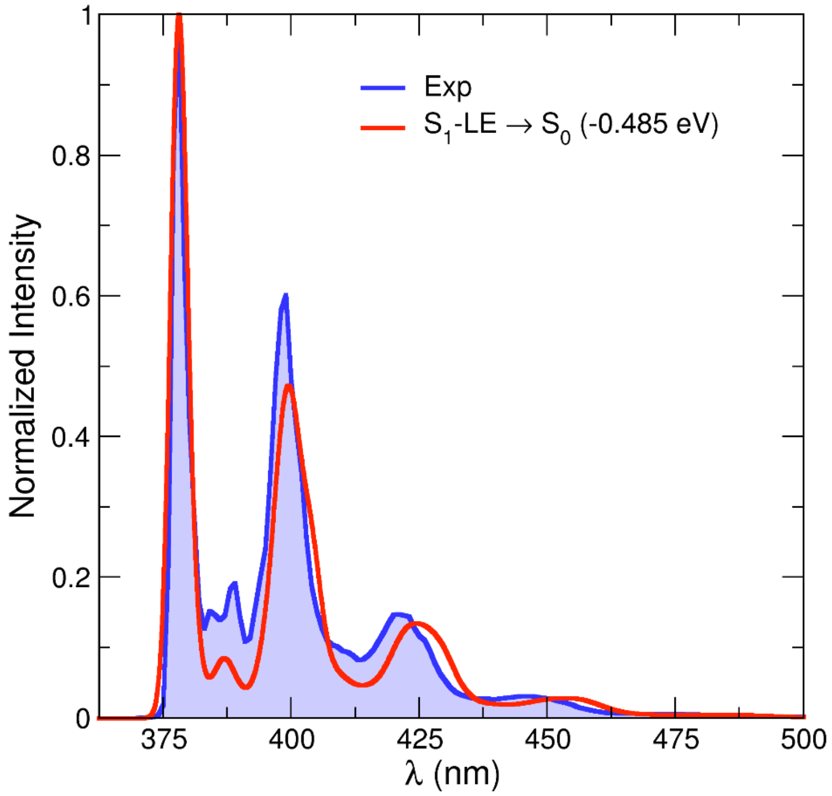

Figure S9: Comparison between the experimental (blue line and area) in methylcyclohexane at 77 K and CAM-B3LYP (red line, rigidly shifted by -0.5 eV) emission spectra. The theoretical emission spectrum only contains the contribution from the S_1-LE state.

Radiative decay and internal conversion rates in hexane solution

In hexane solution, where our steady state (temperature-dependent LE:CT band ratios) and time-resolved fluorescence experiments (monoexponential decays with identical lifetimes at any temperature) suggest that the LE and CT states of **1** are in thermodynamic equilibrium, and equations 1 and 2 apply at time *t* during a time-resolved measurement, the ratio of the two states is a constant, that is:

$$K = \frac{[\mathrm{CT}]_t}{[\mathrm{LE}]_t} = \frac{[\mathrm{CT}]_0 e^{-t/\tau_{\mathrm{CT}}}}{[\mathrm{LE}]_0 e^{-t/\tau_{\mathrm{LE}}}} = \frac{[\mathrm{CT}]_0}{[\mathrm{LE}]_0} e^{t(\frac{1}{\tau_{\mathrm{LE}}} - \frac{1}{\tau_{\mathrm{CT}}})} = const. \qquad \text{(Eq S1)}$$

which indicates that the exponential term equals 1 and $\tau_{\mathrm{CT}} = \tau_{\mathrm{LE}}$ as K is a time-independent constant. This is a prerequisite for a thermodynamically equilibrated dual-excited states system.

The total radiative decay rate (k_r) in a thermodynamically equilibrated system can be calculated from the absolute fluorescence quantum yields of both emission bands (Φ = 0.11) and lifetimes measured in hexane at room temperature:

$$k_r = k_{r,\mathrm{LE}} + k_{r,\mathrm{CT}} = \Phi / \tau = 4.89 \times 10^6 \ \mathrm{s}^{-1} \qquad \text{(Eq S2)}$$

Fitting equation 6 to the Stevens-Ban plot (Figure 3b) yields

$$\ln \frac{k_{r,\mathrm{CT}}}{k_{r,\mathrm{LE}}} = -3.23 \ (7) \qquad \text{(Eq S3)}$$

as the intersection with the y axis for $T \to \infty$

By combining equations S2 and S3, we evaluated the radiative decay rate constant of the LE band $k_{r,\mathrm{LE}}$ =

4.70 (1) x 10^6 s^{-1}, being ca. one order of magnitude faster than that of the CT band ($k_{r,CT}$ = 1.9 (1) x 10^5 s^{-1}). However, both rate constants are overall relatively small because they refer to (almost) forbidden transitions, from the pyrene LE state because it is symmetry forbidden and the CT state because of the small Franck-Condon factors caused by strong structural distortion.

Thermodynamic equilibrium in solvents with different polarities

The band ratio changes in hexane with dioxane additive, according to equation 5,

$$\ln\frac{I_{CT}'}{I_{LE}'} - \ln\frac{I_{CT}}{I_{LE}} = \ln\frac{k_{r,CT}'}{k_{r,LE}'}K' - \ln\frac{k_{r,CT}}{k_{r,LE}}K \qquad \text{(Eq S4)}$$

where apostrophes represent dioxane added, and due to the fact that the radiative decay of pyrene is not much affected by solvent polarity, that is, $k_{r,LE} \approx k_{r,LE}'$, equation S4 can be written as,

$$\ln\frac{I_{CT}'}{I_{LE}'} - \ln\frac{I_{CT}}{I_{LE}} = \ln K' - \ln K + \ln\frac{k_{r,CT}'}{k_{r,CT}} = \frac{\Delta G - \Delta G'}{RT} + \ln\frac{k_{r,CT}'}{k_{r,CT}}$$

and thus $\ln\frac{k_{r,CT}'}{k_{r,CT}} = \ln\frac{I_{CT}'}{I_{LE}'} - \ln\frac{I_{CT}}{I_{LE}} - \frac{\Delta G - \Delta G'}{RT}$ (Eq S5)

The LE:CT band ratio changes from 1:2.6 to 1:16 by adding 10% of dioxane into the hexane solution of **1**, indicating that $k_{r,CT}$ dramatically decreases by factor 0.019 with 10% dioxane added, according to equation 11. This is probably because a more polar solvent further stabilizes the CT state and distorts the molecular geometry from the ground state geometry and decreases the Franck-Condon factors, i.e., the degree of overlap of the nuclear wave functions for the ground state and excited state of **1**.

Single-crystal X-ray diffraction and solid-state emission

Table S1. Details of in-house single-crystal X-ray diffraction data and structure refinements of compound **1**, 1-(pyren-2-yl)-*o*-carborane.

Data	Compound **1**
CCDC number	1863611
Empirical formula	$C_{18}H_{20}B_{10}$
Formula weight / g·mol^{-1}	344.44
T / K	100(2)
λ / Å, radiation	0.71073, Mo-K_{α}
Crystal size / mm^3	0.09×0.12×0.34
Crystal color, habit	colorless, needle
Crystal system	triclinic
Space group	$P\bar{1}$
a / Å	7.1379(15)
b / Å	19.799(4)
c / Å	27.542(5)
α / °	110.801(5)
β / °	90.566(9)
γ / °	90.757(7)
Volume / Å^3	3637.8(13)
Z	8
ρ_{calc} / g·cm^{-3}	1.258
μ / mm^{-1}	0.063
Minimum/maximum transmission	0.593/0.746
F(000)	1424
θ range / °	2.057–26.022
Index ranges	$-8 \leq \eta \leq 8$,<br>$-24 \leq \kappa \leq 22$,<br>$0 \leq \lambda \leq 33$
Reflections collected	72311
Unique reflections	14263
R_{int}	0.1292
Observed reflections [I>2σ(I)]	10789
Parameters / restraints	1010 / 0
GooF on F^2	1.052
R_1 [I>2σ(I)]	0.1028
wR^2 (all data)	0.2695
Max. / min. residual electron density / e·Å^{-3}	0.446 / –0.370

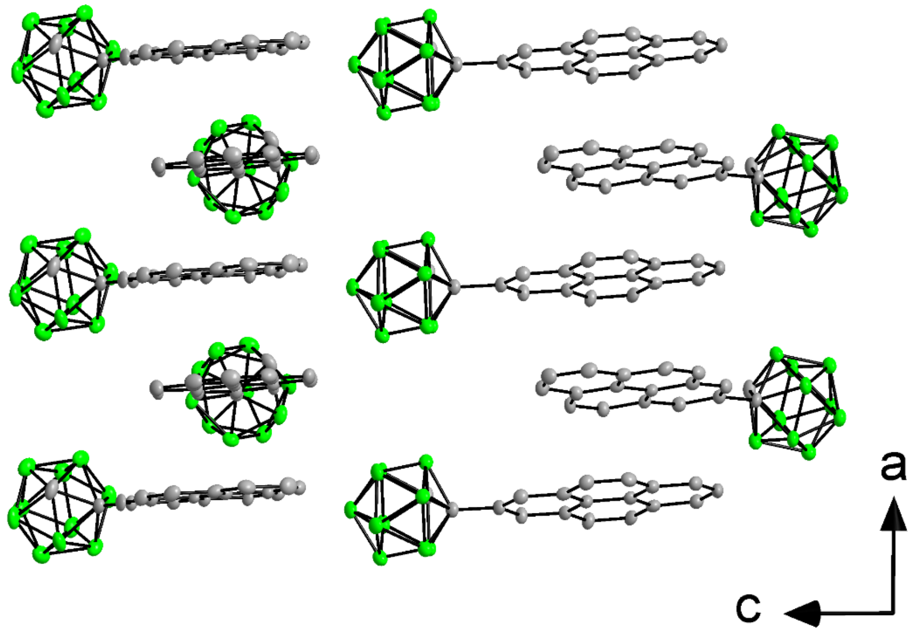

Figure S10. The π-stacking of the *1-(pyren-2-yl)-o-carborane* molecules parallel to the *a* axis is shown for the two different stacks in a projection along the *b* axis.

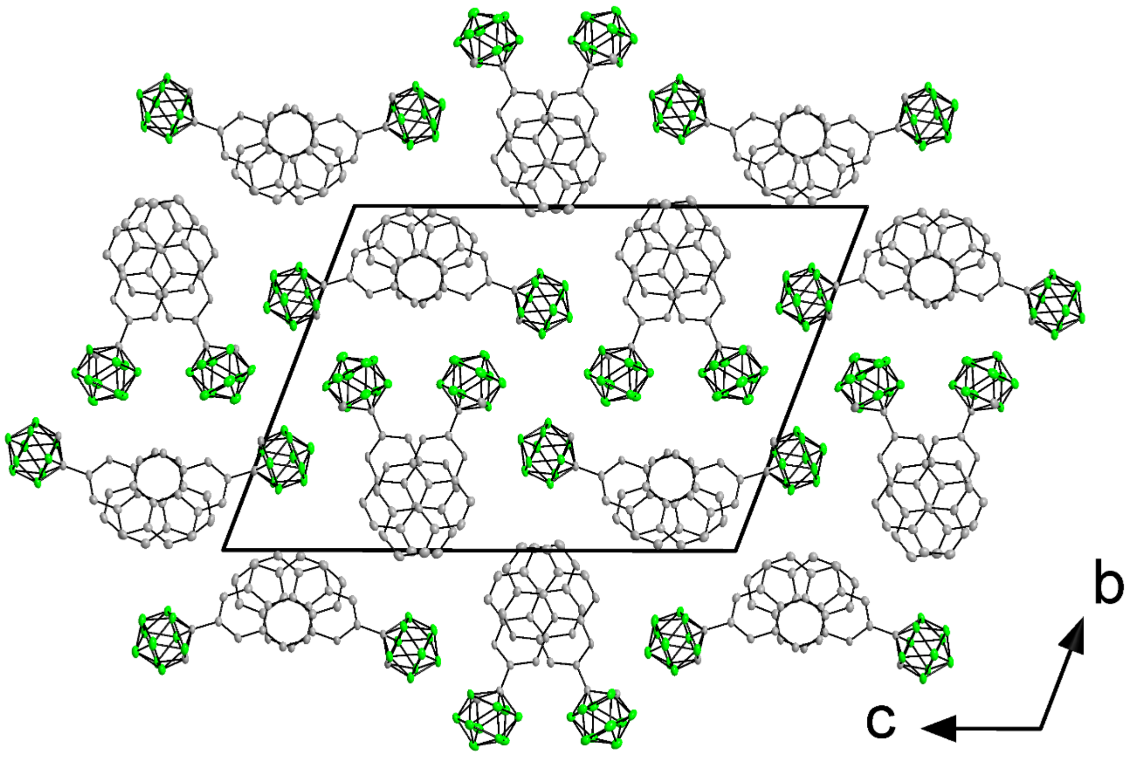

Figure S11. Crystal structure of *1-(pyren-2-yl)-o-carborane* projected along the *a* axis. The unit cell is shown by bold black lines. Molecules pack in alternating stacks parallel to the *a* axis. Two different types of stacks are observed; one of them shows a large rotation angle between the two stacking pyrene orientations of 142.3(3)°, as is defined by the angle between the two C–C bonds connecting the pyrene and the carborane moieties. The other stack shows a smaller rotation angle of 44.5(3)° between stacked molecules.

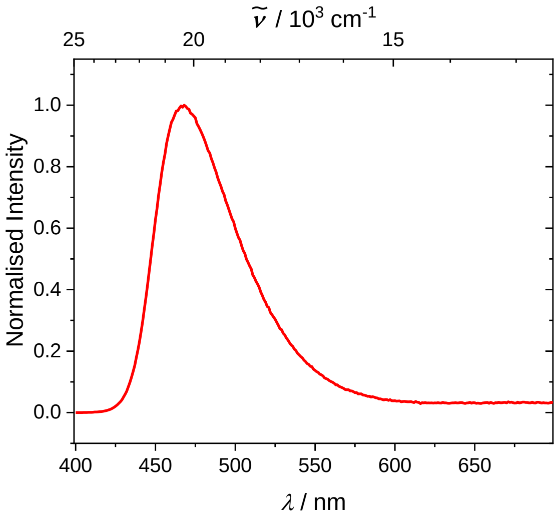

Figure S12. Emission spectrum of **1** in the crystalline state.

Single-crystal synchrotron X-ray diffraction: Small single crystal plates of 1-(pyren-2-yl)-*o*-carborane (diameter ca. 30–60 µm, thickness ca. 10 µm) were selected and glued on MiTeGen sample holders using a 2-component glue. Intensity data were collected at 100 K on three single crystals at the Small Molecule Single Crystal Diffraction beamline I19 at Diamond Light Source (UK). A three-circle diffractometer equipped with a Pilatus 2M detector from DECTRIS and synchrotron radiation with a wavelength of 0.6889 Å were used. The sample-to-detector distance was 160 mm. A total of 3450 exposures (0.2° /frame rotation, 0.196 s exposure time) were collected for each of the crystals. Data reduction and empirical absorption correction were performed with the *CrysAlis^{Pro}* software (version 1.171.39.32e).[1]. The crystal structure was solved in space group $P\bar{1}$ from the best one of the data sets using the intrinsic phasing method (SHELXT,[2]) and Fourier expansion technique. Due to a low quality of the fit, all atoms were refined with isotropic displacement parameters and hydrogen atoms 'riding' in idealized positions, by full-matrix least squares against F^2 of all data. The SHELXL[3] software and the SHELXLE graphical user interface[4] were used. The C−C bond distances of the pyrene moieties were restrained to ideal values. A two-fold rotation twin with twin matrix [1 0 0, 0 -1 0, 0 0 -1] was refined with a twin component of about 25.8%. The carborane moieties were refined with only one carbon atom and 11 boron atoms each as the position of the second carbon atom could not be reliably distinguished among the carbon-surrounding boron positions either due to the low quality of the data or disorder. The final residual values converged to R1 = 0.345 for reflections with $F_o > 4\sigma(F_o)$ and $wR^2 = 0.653$ for all reflections. This led to the conclusion that something was wrong either with the structural model, which looked reasonable with respect to the molecular packing, or with the data. It could be that the intensities obtained were incorrect due to the choice of inappropriate data collection parameters. The diffraction data from all three crystals showed diffuse reflection streaks along the reciprocal $\vec{b}^*$ direction in every row with odd *l* indices (Figure S13).

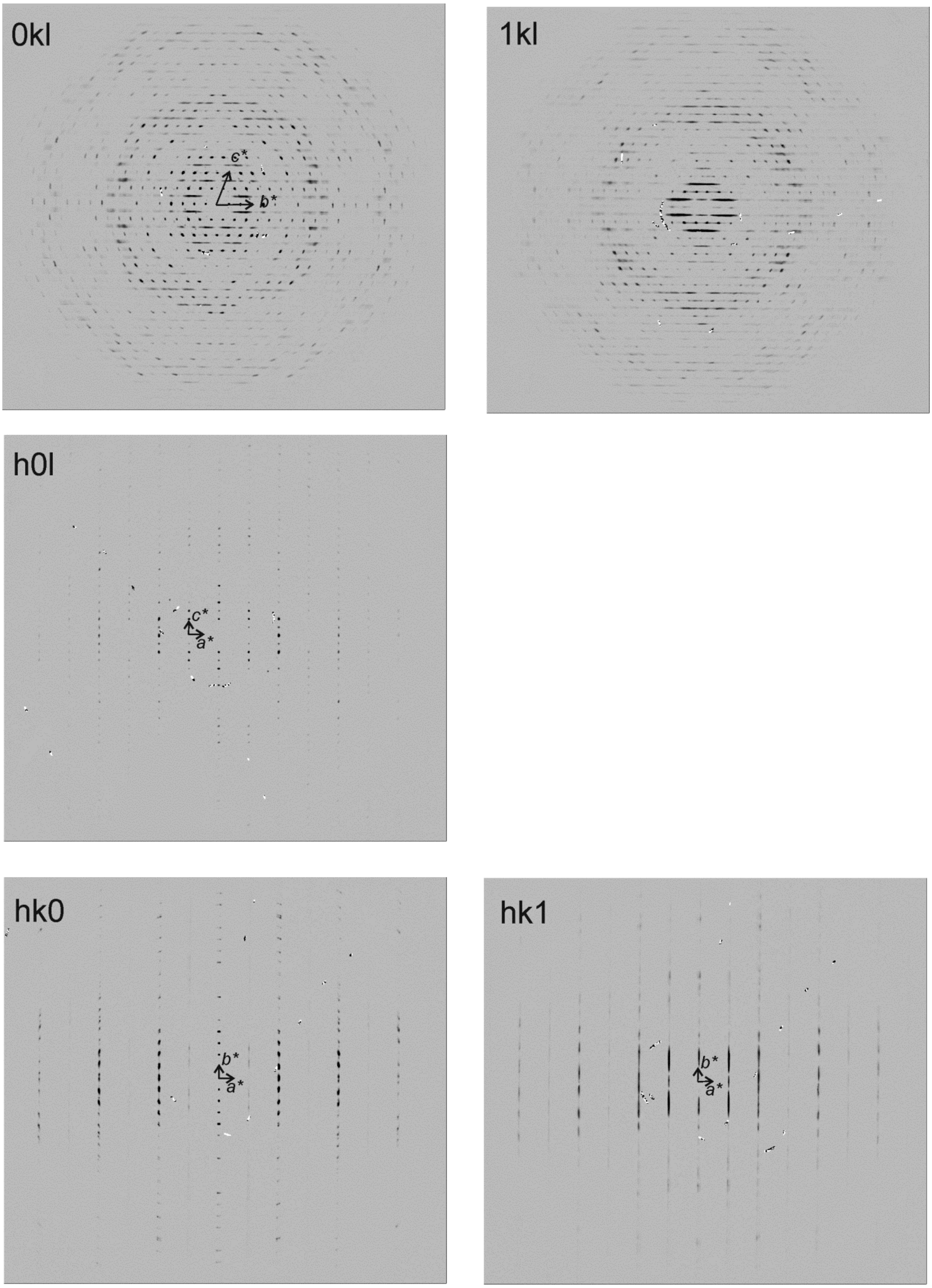

Figure S13. Reciprocal layers of the single-crystal X-ray diffraction data reconstructed from the synchrotron data collection using *CrysAlis*^{Pro} software. Diffuse scattering is observed in b^* direction in every row with odd l indices as can be seen in the (0kl), (1kl), and (hk1) layers.

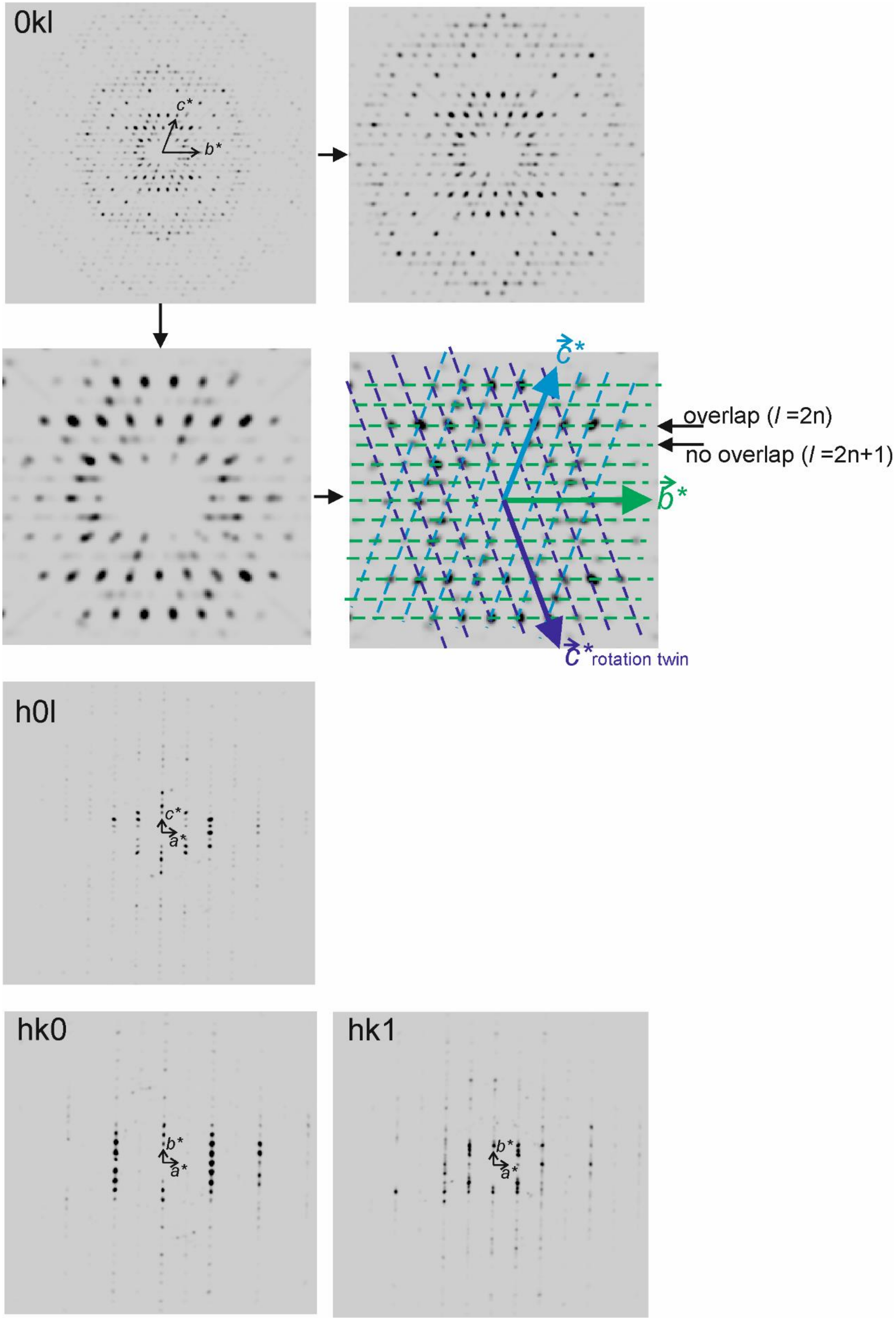

Figure S14. Reciprocal layers of the single-crystal X-ray diffraction data reconstructed from the in-house data collection using CrysAlis^{Pro} software. The 180° rotation twin around the b* reciprocal axis can explain the additional Bragg reflections in $\vec{b}^*$ direction in every row with odd l indices as can be seen in the (0kl) and (hk1) layers.

NMR Spectra

^{1}H NMR spectrum of 1

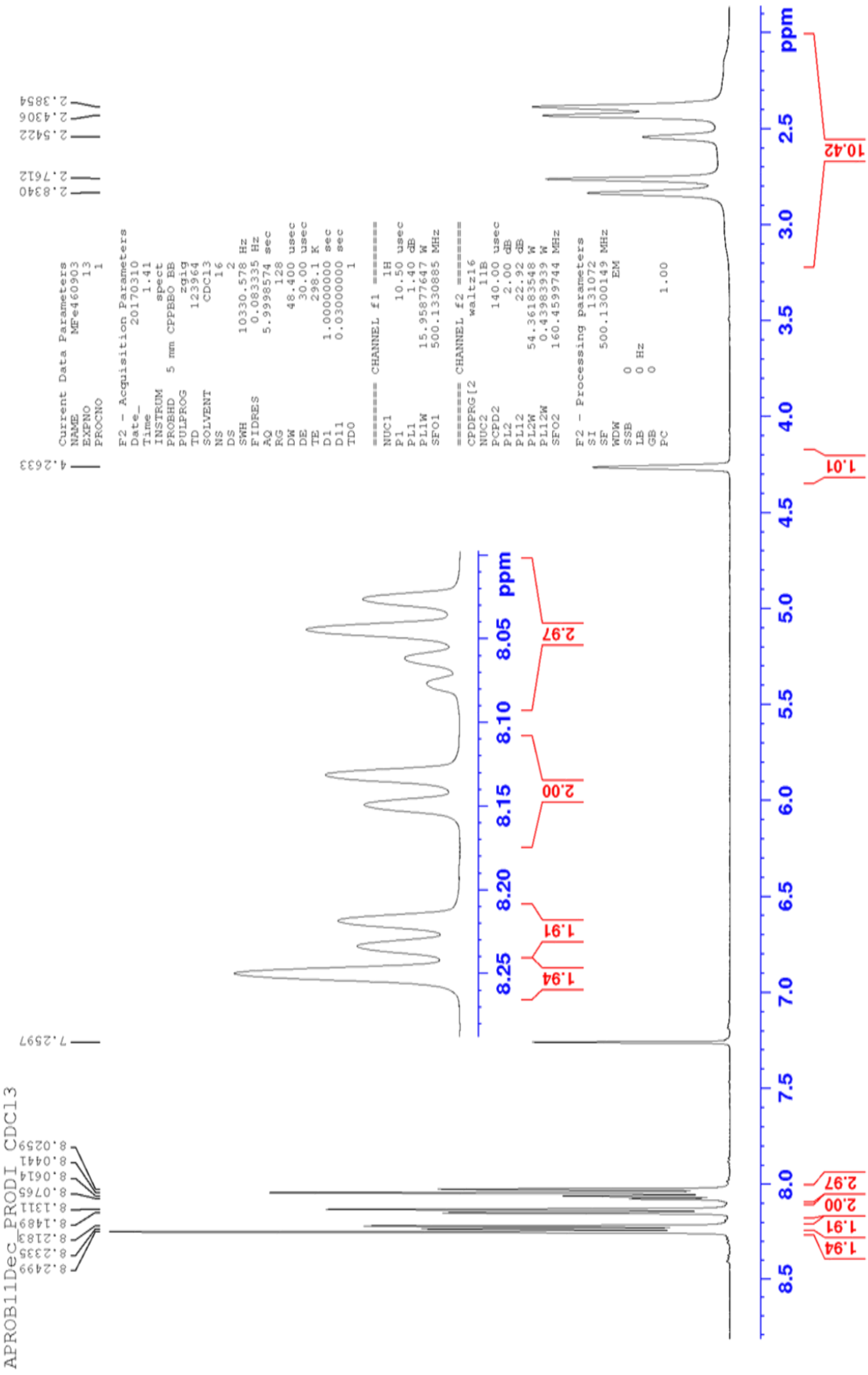

^{13}C NMR spectrum of 1

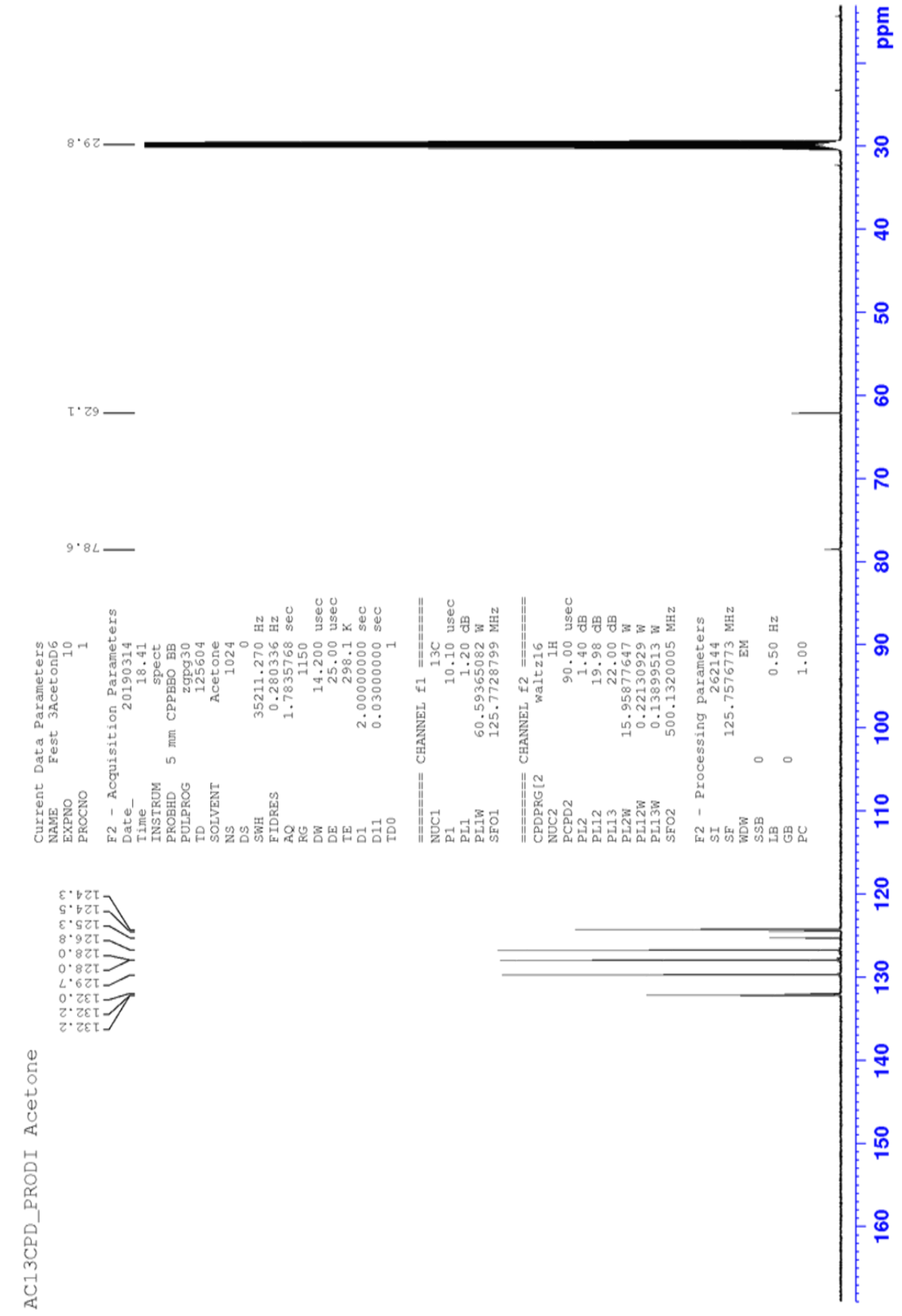

^{11}B NMR spectrum of 1

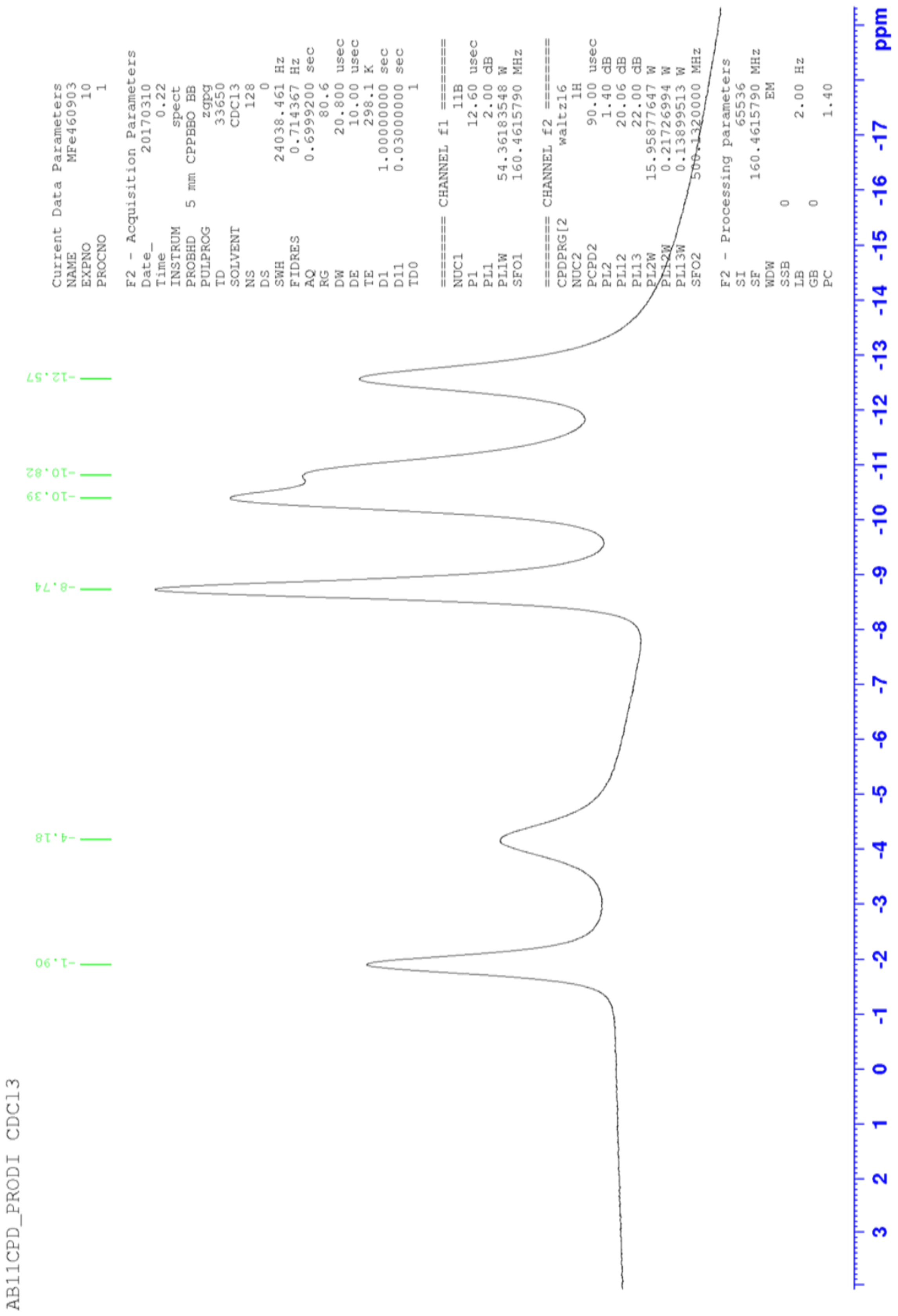

Cartesian coordinates of all optimized states

S_1-M

C	0.000000	0.000000	0.000000
C	0.000000	0.000000	2.617072
B	1.046442	0.000000	1.338999
B	1.017350	-1.249397	-0.224577
B	-0.758061	-1.421155	-0.227859
B	-1.026508	-0.197228	1.337709
B	-0.769450	-1.384760	2.864694
B	1.016140	-1.214428	2.867331
B	1.590484	-1.873563	1.320420
B	0.258965	-2.688081	0.448971
B	-1.205562	-2.141306	1.317555
B	0.255015	-2.668317	2.216474
H	-0.081988	0.850386	3.281311
H	1.829654	0.891055	1.310374
H	1.758493	-1.179425	-1.147286
H	-1.496351	-1.493912	-1.152603
H	-1.964874	0.528486	1.309142
H	-1.508012	-1.460600	3.789094
H	1.753838	-1.150058	3.793304
H	2.726207	-2.221257	1.317839
H	0.358365	-3.720672	-0.131470
H	-2.255013	-2.697657	1.313567
H	0.352570	-3.694676	2.807039
C	-0.107978	1.138895	-0.898206
C	-0.086431	0.948649	-2.290461
C	-0.235659	2.442482	-0.390375
C	-0.188418	2.020272	-3.166967
C	-0.340329	3.539956	-1.234488
C	-0.167979	1.843191	-4.596397
C	-0.317525	3.337053	-2.642846
C	-0.471954	4.883181	-0.731327
C	-0.268723	2.899169	-5.432015
C	-0.423337	4.447145	-3.524309
C	-0.572450	5.936522	-1.570319
C	-0.400327	4.242054	-4.929850
C	-0.552221	5.760949	-2.999194
C	-0.505647	5.346293	-5.776803
C	-0.654742	6.836128	-3.882865
C	-0.631417	6.628147	-5.256291
H	0.012056	-0.057617	-2.683804
H	-0.069216	0.834859	-4.986484
H	-0.252076	2.753898	-6.508069
H	-0.488407	5.193812	-6.851899
H	-0.712255	7.475715	-5.929351
H	-0.753376	7.842018	-3.485636
H	-0.671336	6.944841	-1.179298
H	-0.488428	5.028624	0.344597
H	-0.252868	2.595513	0.683842

S1-LE

C	-2.348700	0.096700	-0.061400
C	-3.151200	-1.109500	0.696000
B	-3.146400	0.413600	1.449800
B	-3.251800	1.537500	0.092100
B	-3.258200	0.615500	-1.419400
B	-3.157500	-1.096800	-1.012400
B	-4.592900	-0.604100	1.427900
B	-4.696600	1.110800	1.021800
B	-4.773100	1.237300	-0.753600
B	-4.711000	-0.402800	-1.434700
B	-4.589700	-1.536100	-0.078600
B	-5.604800	-0.093900	0.072900
H	-2.449800	0.564200	2.391800
H	-2.525000	-1.856400	1.164700
H	-2.678600	2.568900	0.163900
H	-2.647700	1.005000	-2.355900
H	-2.482100	-1.881200	-1.580200
H	-4.908600	-1.127600	2.442100
H	-5.208300	1.895600	1.749500
H	-5.347700	2.124900	-1.292200
H	-5.232800	-0.712100	-2.454200
H	-4.908700	-2.676400	-0.067200
H	-6.786600	-0.181300	0.131200
C	-0.854000	0.060200	-0.047700
C	-0.154000	-1.163100	-0.046900
C	-0.117400	1.259400	-0.042900
C	1.244800	-1.214500	-0.025900
H	-0.694400	-2.102600	-0.084300
C	1.281900	1.266200	-0.029200
H	-0.638800	2.208300	-0.052100
C	2.002300	0.014500	-0.015400
C	1.966000	-2.439800	-0.020000
C	2.038800	2.469300	-0.025100
C	3.386200	-0.006100	0.002000
C	3.337900	-2.461100	-0.000400

H	1.409400	-3.372400	-0.031300
C	3.410600	2.450800	-0.006700
H	1.509000	3.417200	-0.036500
C	4.099700	-1.261400	0.011900
C	4.137300	1.228000	0.008000
H	3.864400	-3.411100	0.004800
H	3.965200	3.384400	-0.003600
C	5.502600	-1.249500	0.030900
C	5.538500	1.175100	0.026800
C	6.218100	-0.048100	0.038700
H	6.034900	-2.196100	0.038700
H	6.098600	2.105400	0.031500
H	7.302100	-0.064600	0.053200

S1-CT

C	2.190911	0.059623	0.106743
C	3.513249	-1.071072	-1.459284
B	3.099495	0.551815	-1.284899
B	3.129445	1.346460	0.381597
B	3.108225	-0.123039	1.424891
B	3.070664	-1.433397	0.124427
B	4.799204	-0.104385	-1.611725
B	4.606735	1.302100	-0.561029
B	4.622913	0.761214	1.144346
B	4.573095	-1.026498	1.092017
B	4.778165	-1.558330	-0.579607
B	5.604191	-0.086518	-0.029836
H	2.499803	1.086602	-2.165047
H	3.102194	-1.646300	-2.278094
H	2.564583	2.370682	0.607681
H	2.526955	-0.236527	2.458548
H	2.449059	-2.429080	0.330614
H	5.305609	-0.008298	-2.682404
H	5.080738	2.338345	-0.902071
H	5.116187	1.383413	2.030677
H	5.022407	-1.699142	1.964065
H	5.267949	-2.610389	-0.835198
H	6.791410	-0.054703	0.039245
C	0.731286	0.083895	0.111005
C	-0.011209	-0.878601	0.801372
C	0.017181	1.067869	-0.579052
C	-1.427054	-0.888595	0.805720
H	0.516788	-1.646827	1.355145
C	-1.397998	1.102760	-0.606539
H	0.567582	1.834061	-1.113488
C	-2.134030	0.112341	0.092169
C	-2.165737	-1.870699	1.506334
C	-2.107780	2.102824	-1.311602
C	-3.541839	0.125644	0.081972
C	-3.541973	-1.862424	1.497530
H	-1.630809	-2.638681	2.055517
C	-3.483912	2.118509	-1.325620
H	-1.550564	2.864522	-1.847206
C	-4.265883	-0.869151	0.788523
C	-4.236660	1.135219	-0.632914
H	-4.091629	-2.625253	2.040289
H	-4.011119	2.893501	-1.873397
C	-5.675444	-0.834693	0.762589
C	-5.646845	1.126710	-0.628377
C	-6.356291	0.152182	0.061520
H	-6.229320	-1.596393	1.303075
H	-6.178344	1.899233	-1.175924
H	-7.440106	0.162206	0.053371

S0 / FC

C	-2.346389	0.095271	-0.040711
C	-3.151811	-1.125876	0.678680
B	-3.150663	0.383508	1.464035
B	-3.245458	1.536727	0.132216
B	-3.230517	0.645299	-1.395906
B	-3.138321	-1.077656	-1.026278
B	-4.597763	-0.633766	1.405729
B	-4.698796	1.090566	1.035305
B	-4.755374	1.254215	-0.736984
B	-4.684331	-0.371238	-1.453246
B	-4.584222	-1.533041	-0.120106
B	-5.596011	-0.092796	0.051167
H	-2.464450	0.511626	2.416674
H	-2.532247	-1.882359	1.140274
H	-2.668234	2.562964	0.234323
H	-2.606695	1.053185	-2.315319
H	-2.452958	-1.847297	-1.601856
H	-4.924400	-1.176835	2.405824
H	-5.218417	1.857849	1.775649
H	-5.323075	2.152468	-1.264862
H	-5.194198	-0.656364	-2.485496
H	-4.904657	-2.672791	-0.136100
H	-6.778306	-0.180047	0.094897
C	-0.837926	0.058009	-0.021658
C	-0.151427	-1.154702	0.000063
C	-0.116726	1.248951	-0.042086
C	1.243073	-1.198448	0.012471
H	-0.690364	-2.096047	-0.014705

C	1.278592	1.248528	-0.036883	C	4.089013	-1.244713	0.026428
H	-0.637582	2.197927	-0.065876	C	4.124399	1.212493	-0.022273
C	1.974176	0.014835	-0.006140	H	3.855445	-3.405887	0.062770
C	1.968609	-2.440877	0.036704	H	3.954153	3.379299	-0.069766
C	2.039844	2.469479	-0.059773	C	5.485675	-1.242124	0.031643
C	3.398831	-0.005952	0.000098	C	5.520454	1.169342	-0.016263
C	3.320211	-2.460907	0.044339	C	6.191465	-0.046239	0.010680
H	1.403212	-3.368220	0.048006	H	6.017772	-2.188858	0.051524
C	3.391444	2.450406	-0.052171	H	6.079621	2.100373	-0.033731
H	1.500505	3.411709	-0.083490	H	7.276725	-0.061792	0.014578

References

1. *CrysAlis^{Pro}* software system, Rigaku Oxford Diffraction, 2017.
2. G. M. Sheldrick, *Acta Crystallogr., Sect. A: Found. Crystallogr.* **2015**, *A71*, 3-8.
3. G. M. Sheldrick, *Acta Crystallogr., Sect. A: Found. Crystallogr.* **2008**, *A64*, 112-122.
4. C. B. Hübschle, G. M. Sheldrick, B. Dittrich, *J. Appl. Cryst.*, **2011**, *44*, 1281−1284.
